# Mother–Child Relationship Quality in the Presence of Maternal Mental Disorders: Do Self‐Report and Behavioural Observation Differ?

**DOI:** 10.1002/cpp.70227

**Published:** 2026-03-07

**Authors:** Anne Jung, Robert Kumsta, Babette Renneberg, Silvia Schneider, Nina Heinrichs

**Affiliations:** ^1^ Department of Psychology, Clinical Child and Adolescent Psychology and Psychotherapy Bielefeld University Bielefeld Germany; ^2^ Department of Psychology, Clinical Psychology and Psychotherapy University of Bremen Bremen Germany; ^3^ Department of Behavioural and Cognitive Sciences, Faculty of Humanities, Education and Social Sciences University of Luxembourg Esch‐sur‐Alzette Luxembourg; ^4^ German Center for Mental Health (DZPG), Partner Site Bochum/Marburg Bochum Germany; ^5^ Department of Education and Psychology, Clinical Psychology and Psychotherapy Freie Universität Berlin Berlin Germany; ^6^ German Center for Mental Health (DZPG), Partner Site Berlin/Potsdam Berlin Germany; ^7^ Clinical Child and Adolescent Psychology, Mental Health Research and Treatment Center Ruhr University Bochum Bochum Germany

**Keywords:** behavioural observation, borderline personality disorder, mental disorder, mother–child interaction, self‐report

## Abstract

Mental disorders affect not only mothers themselves but also their children and partners. Borderline personality disorder (BPD) in particular is thought to impact the mother–child relationship, but comparisons with mothers with other mental disorders are scarce. Many studies use questionnaires without examining if self‐report corresponds to observable behaviour. We assessed the perceived mother–child relationship using the Parenting Relationship Questionnaire and the Child Relationship Behaviour Inventory in three groups: (1) mothers with BPD, (2) mothers with anxiety and/or depression and (3) mothers without mental disorders with preschool children. Additionally, mother–child interactions during free‐play and structured tasks were video‐recorded and coded using the Coding Interactive Behaviour system. Compared with mothers without mental disorders, both clinical groups perceived their relationship with the child, their own parenting skills and their children's behaviour as less positive. Mothers with BPD felt less confident and more frustrated than those with anxiety and/or depression. No significant group differences emerged in observed behaviour (e.g., sensitivity, intrusiveness), and correlations between self‐report and observation were low. Overall, mothers with BPD face similar challenges as mothers with anxiety or depressive disorders, but they experience particular distress when it comes to relating to and controlling their child's affect. While mothers in both clinical groups are able to foster positive relationships with their children in a controlled laboratory setting, they find it difficult to maintain these skills permanently (e.g., during negative child affect).

**Trial Registration:** DRKS‐ID: DRKS00020460

## Summary

- Mothers with BPD, as well as those with anxiety or depression, perceive their parenting and the relationship with their child more negatively than mothers without mental disorders.
- Mothers with BPD in particular report high frustration in parenting.
- In terms of observed parenting behaviour, mothers with BPD did not differ from those with anxiety or depression, or from those without mental disorders.
- Despite self‐reported difficulties, all groups—including clinical—were able to foster positive interactions with their children in controlled observation settings.

## 1 Introduction

Many children grow up with a parent affected by mental disorders, estimated at one in five worldwide (Maybery and Reupert 2018), and such parental mental illness has been identified as the most common adverse childhood event (Marie‐Mitchell and Kostolansky 2019). Parental mental disorders shape the social environment in which children grow up by impeding appropriate parenting skills and reducing confidence in parenting (Derella and Milan 2021; Goodman et al. 2022). Parenting can be divided into two broad dimensions: ‘one pertaining to parenting as behavioral or psychological control and child management […] and the other related to parenting as […] parent‐child emotional bond or attachment (i.e., emotional relationship)’ (Cummings and Cummings 2002, 52). Many parenting questionnaires relate to dysfunctional (and some also to functional) parenting practices, including items relating to how parents set and monitor rules or how they react upon rule‐breaking behaviour. While many of the parenting self‐report measures relate to control and child management, those that relate to positive practices cover positive forms of child management (e.g., clear instructions and praising) or the emotional parent–child relationship (e.g., enjoying spending time together). In this study, we focus on parenting as a reflection of the parent–child relationship (i.e., relational/emotional focus) as opposed to a focus on controlling or scaffolding parenting skills and parenting styles (i.e., an educational, socializing or scaffolding focus) (cp. Lorence et al. 2025).

Parental mental disorders are linked with various adverse child outcomes (see Stein et al. 2014) and are a known risk factor for the development of psychopathology in children (Ayano et al. 2021), with parenting acting as a mediator of this transgenerational transmission (Stein et al. 2014). How the parent feels and thinks influences their parenting and relationship behaviour (Taraban and Shaw 2018). More dysregulated child characteristics are also associated with increased parenting stress which, in turn, can shape child characteristics through daily parent–child interactions (McQuillan and Bates 2017). Characteristics of parent and child therefore mutually affect each other's behaviour, cognitions, and emotions and contribute to the parent–child relationship.

A group that consistently reports high levels of parenting stress (Newman et al. 2007) and difficulties in parenting and the parent–child relationship (Florange and Herpertz 2019) are parents with borderline personality disorder (BPD). Difficulties in emotion regulation, unstable relationships, and fears of abandonment are the core DSM‐5 features of BPD (American Psychiatric Association 2013) that are likely to spill over into the parent–child relationship (Renneberg and Rosenbach 2016), increasing the risk for dysfunctional mother–child interactions and low relationship quality (Petfield et al. 2015). Compared with mothers without mental disorders, the perception of own and others' emotional needs and appropriate reactions to them is often difficult for mothers with BPD (Petfield et al. 2015; Renneberg and Rosenbach 2016). Their interactions are described as involving more role reversal, boundary confusion and a reluctance to promote autonomy (Eyden 2021; Eyden et al. 2016; Petfield et al. 2015). At a cognitive level, mothers with BPD struggle with feelings of shame and uncertainty (Kasiviswanathan et al. 2025). Simultaneously, they are highly concerned for their children's safety and care deeply for them (Eyden et al. 2016), challenging the assumption of solely negative or dysfunctional mother–child relationships. The majority of the studies presented so far refer to mothers' self‐report of parenting skills; only very few studies include behavioural observation. Those available showed that mothers with BPD are associated with less sensitivity and less engagement, less structured and more ‘intrusively insensitive’ (Hobson et al. 2005, 329) behaviour compared with a control group (Eyden et al. 2016; Newman et al. 2007). Mothers with BPD are assumed to oscillate between overinvolved, intrusive, hostile (‘too much’) and disengaged, withdrawn (‘too little’) behaviour (Stepp et al. 2012).

Most often, mothers with BPD are compared with healthy controls and considerably fewer studies have investigated whether these parenting aspects are particularly challenging for parents with BPD (disorder‐specific hypothesis) or whether they are challenging for parents with any mental disorder (transdiagnostic hypothesis). For example, it is assumed that symptoms associated with depressive disorders, such as a lack of drive or dysphoric affect, hinder parents from interacting sensitively with their children (Field 2010). Reviews do in fact suggest a link between depression and reduced sensitivity as well as compromised caregiving activities like breastfeeding and sleep routines (Field 2010). Anxiety disorders, e.g., social anxiety disorder, have also been reported to be associated with specific parenting (e.g., a controlling style low in emotional warmth; Garcia et al. 2021). Higher levels of depressive symptoms are linked to experiencing self‐blame and intense feelings of guilt (Derella and Milan 2021). At a cognitive level, mothers with anxiety disorders see their children more negatively in terms of their ability to self‐regulate and effortfully control themselves (Miller et al. 2021). Higher levels of anxiety and depressive disorders are furthermore linked to perceiving their children as more dominant and less positive (Lefkovics et al. 2018). With regard to self‐reported parenting, symptoms of general anxiety disorder were associated with more overinvolved parenting (Möller et al. 2015). Similar results were reported from behavioural observation studies (anxiety: Nicol‐Harper et al. 2007; depression: Bernard et al. 2018). Compared with healthy controls, anxiety as well as depressive disorders are associated with more intrusive, critical, or hostile dyadic behaviour (Azak and Raeder 2013; Schneider et al. 2009). Results are, however, inconsistent, as some researchers have found no significant differences in terms of dyadic behaviour between mothers with and without a depression (Healy et al. 2016).

When a clinical comparison group was examined, mothers with BPD were most often compared with mothers with depression. Tentative first results indicate a specificity of this disorder: Compared with depressive disorders, BPD was associated with more disrupted affective communication in the form of contradictory signals, like a threatening message in a sweet voice, as well as more frightened and disoriented behaviour, such as freezing or sudden shifts in voice tone (Hobson et al. 2009). Additionally, BPD was associated with fewer instances of imitating the infant's behaviour, as well as less touching and playing games, compared with depression and no mental disorder (White et al. 2011). A comparison of mothers with BPD (*n* = 8), mothers with depression, and a healthy control group revealed that, while mothers with depression interacted less sensitively with their children, mothers with BPD displayed higher levels of hostility in direct interactions (Kluczniok et al. 2018). The authors suggested that these mental disorders are associated with qualitatively different patterns of emotional availability, supporting a disorder‐specific hypothesis. Mothers with BPD, depression, and mothers without mental disorders are currently also examined with regard to self‐reported parenting, focusing on parenting strategies (i.e., educational focus), using the same sample as the present study. Again, the authors found disorder‐specific group differences, with the BPD group reporting more maladaptive parenting strategies (Rosenbach et al. 2025, manuscript submitted for publication).

### 1.1 The Current Study

The current study was part of a multicentre study (‘ProChild’) that was funded by the German Federal Ministry of Education and Research (01KRI805C). It comprises five subprojects that aimed to evaluate a parenting programme for mothers with BPD (Subproject 1) while examining child development (Subproject 2), family climate (Subproject 3), epigenetics (Subproject 4) and the collaboration between childcare institutions, mothers with BPD and their psychotherapists (Subproject 5). This study aimed to examine concrete, observable relationship behaviour and relational cognitions in mothers with BPD, mothers with anxiety and/or depressive disorders (clinical comparison group) and a nonclinical comparison group. Aspects of parenting that reflect an educational focus are part of Subproject 1 and published elsewhere (Rosenbach et al. 2025, manuscript submitted for publication).

The present study aimed to compare parent–child relationship aspects assessed via behavioural observations and self‐report. We observed dyadic behaviour in both free‐play and structured tasks, as context often elicits different behaviour. Some studies reported more favourable dyadic behaviour in free‐play situations (Kwon et al. 2013), others did so in structured tasks (Dittrich et al. 2017). Our approach aimed to (1) examine if difficulties in the mother–child relationship quality assessed via self‐report translate to observed behaviour and vice versa; (2) to examine if, in observed behaviour, these associations are similar across different contexts; and (3) if and how mental disorders are associated with characteristics of the parent–child relationship. We decided to focus on maternal (not paternal) mental disorders, as our primary interest is in supporting individuals with BPD with small children (who are predominately female). The anxiety and/or depressive disorder comparison group (ad/D) was chosen due to their high prevalence rates (Jacobi et al. 2004). We predicted that mothers with BPD would experience more relationship difficulties than mothers in both other groups, hence supporting the hypothesis of a disorder‐specificity. Moreover, we expected poorer outcomes for mothers in the ad/D group compared to the CON group.

## 2 Methods

The present article reports cross‐sectional results from Subproject 3, specifically aspects of the mother–child relationship (see Jung et al. 2025 for information on the mothers' mental representations). In 2020, Subproject 3 was registered in the German Clinical Trial Register (ID: DRKS00020460), and ethical approval was obtained by the ethics committee of the DGPs.

### 2.1 Procedure

Between 2019 and 2024, data were collected in Berlin, Bochum, Bremen and Bielefeld. We recruited mothers of children between 6 months and 6 years via psychotherapists, kindergartens and social media into three groups: (1) BPD, (2) anxiety disorder or depression or both (ad/D) or (3) no mental disorders in the last 7 years (CON). Mothers were checked for eligibility (e.g., fulfilling respective criteria for a mental disorder or for none mental disorder at all; mothers with BPD were required to have either completed BPD‐related treatment or to be currently in treatment). Exclusion criteria were acute risk of child maltreatment, acute suicidality, acute substance dependence, psychotic symptoms or diagnosed maternal mental retardation. In the control groups (ad/D, CON), a lifetime diagnosis of BPD was considered as an exclusion criterion. Mental disorders were assessed via the Structural Clinical Interview for DSM‐5 disorders (First, Williams, Karg, et al. 2019; First, First, Williams, Smith Benjamin, et al. 2019). After informed consent and eligibility confirmation, mothers received questionnaires and visited the university lab for observation. During the session, mother–child interactions were video‐recorded in two tasks: free‐play with standardized toys (e.g., tea set, building bricks; 12 min with the first 2 min as warm‐up), followed by an 8‐min structured task (feeding for children younger than 3 years; collaborative puzzle for older children). Each family received a financial compensation after the assessments; children were given a present. Each group was assessed before the intervention, followed by a second (after intervention; only for BPD and CON) and third assessment (after 6 months; only for BPD and CON). The present data are from the first assessment.

### 2.2 Participants

Three hundred fifty‐one families were enrolled in the study (*n*_BPD_ = 178, *n*_AD/D_ = 78, *n*_CON_ = 90). Adequate sample sizes per group were preidentified via an a priori power analysis. Due to technical difficulties in the video recordings or dropouts after inclusion, data from *n* = 320 dyads were available for the present research questions with at least one of the outcome measures available. Across all three groups, the mothers were 34 years old on average (5.93 SD), and the children were mostly toddlers with an average age of 3 years (1.85 SD; see Table 1 for group‐wise information).

**TABLE 1:** Sociodemographic data per group.

	BPD	ad/D	CON	Group comparison
*N*	158	66	96	
Mothers				
Age: mean (SD)	**32.80 (6.21)**	**34.24 (5.49)**	**34.56 (5.60)**	*F*(2, 317) = 3.14, *p* = 0.045
Education low: *n* (%)	**88 (55.70%)**	**18 (27.27%)**	**10 (10.42%)**	Kruskal Wallis χ2(2) = 55.70, *p* < 0.001
Psychotherapy (outpatient) during lifetime: *n* (%)	**152 (96.20%)**	**61 (92.42%)**	**30 (31.25%)**	All groups: χ ^2^(2) = 150.25, *p* < 0.001 BPD vs. ad/D: χ ^2^(1) = 0.729, *p* = 0.393
Psychotherapy (inpatient) during lifetime: *n* (%)	**125 (79.11%)**	**27 (40.91%)**	**3 (3.13%)**	All groups: χ ^2^(2) = 139.95, *p* < 0.001 BPD vs. ad/D: χ ^2^(1) = 29.43, *p* < 0.001
Children				
Age: mean (SD)	**3.54 (1.94)**	**3.38 (1.76)**	**2.84 (1.69)**	*F*(2, 317) = 4.39, *p* = 0.013
Girls: *n* (%)	79 (50.00%)	35 (53.03%)	56 (58.33%)	χ ^2^(2) = 1.67, *p* = 0.435
Clinical diagnosis: *n* (%)	**53 (33.54%)**	**26 (39.39%)**	**13 (13.54%)**	χ ^2^(2) = 16.71, *p* < 0.001

*Note:* BPD: mothers with borderline personality disorder, ad/D: mothers with an anxiety disorder, depression or both, CON: mothers without any mental disorder. Child age is displayed in years. Child clinical diagnosis: 2 missing values. Yates' correction was applied to the χ ^2^ test for cells with fewer than 5 observations.

### 2.3 Measures

#### 2.3.1 Maternal Mental Disorder: Structural Clinical Interview for DSM‐5 Disorders

Maternal mental disorders were assessed using two semistructured interviews, the Structured Clinical Interview for DSM‐5—Clinical Version (SCID; First, Williams, Karg, and Spitzer (2019)) and the SCID for Personality Disorders (SCID‐PD; First, Williams, Smith Benjamin, and Spitzer (2019)), including modules for affective, anxiety, psychotic and substance use disorders (current diagnoses) and BPD (assessed as a lifetime diagnosis). Mothers who met the criteria for BPD (assessed with the SCID‐PD), either in their lifetime or currently, were assigned to the BPD group. Mothers with remitted anxiety or depression (assessed with the SCID) were included in the ad/D group if criteria were met within the past 2 months. Interviewers received initial training and ongoing supervision; all interviews were video‐recorded, with 20% independently rated by a senior clinician.

#### 2.3.2 Perception of Mother–Child Relationship and Child Behaviour: Parenting Relationship Questionnaire (PRQ) and Child Relationship Behaviour Inventory (CRBI)

These two questionnaires capture mother–child relationship quality from different angles and are both tailored to children aged 2 and above.

The PRQ (Kamphaus and Reynolds 2015) assesses how parents perceive the parent–child relationship on seven scales, using a 4‐point Likert scale (0 *Never* to 3 *Almost always*). We used the PRQ‐Preschool version for children between two and 5 years (60 items) and for older children the PRQ‐Child and Adolescent version (87 items) translated (and back‐translated) in a German version. The PRQ is divided into five scales for all age groups, as well as two additional scales for older children only. For the present study, we were only interested in those scales available across the total child age range and reflecting a relational/emotional focus (Attachment, Involvement and Relational Frustration; additional information on each scale is provided in the Supporting Information). For each scale separately, sum scores are calculated and transformed into *T*‐scores (provided by the US manual) according to child's age. After the transformation, we collapsed the scale values for younger and older children. The PRQ previously showed good internal consistency (Cronbach's α= 0.82–0.94; Kamphaus and Reynolds 2015). In the present study, Cronbach's α was 0.89–0.94.

As a second measure of maternal perception of the relationship, especially focusing on the perception of how children interact within this relationship, we used the CRBI (Briegel et al. 2019) for children aged two and above. The inventory consists of the Child Relationship Development Questionnaire (CRDQ), assessing positive relationship‐relevant child behaviours (e.g., ‘Approaches me to spend time together’, 14 items), and the Child Relationship Checklist (CRC), assessing negative child behaviours (e.g., ‘Disturbs me on purpose when I need a rest’; 14 items). For both questionnaires, mothers rated the frequency on a 7‐point Likert scale (1, *Never* to 7, *Always*). Sum scores are separately computed, with higher scores reflecting a higher amount of perceived positive or negative child behaviours, respectively. In line with the suggestion in Briegel et al. (2019) that a ratio of positive (CRDQ) to negative (CRC) perceived child behaviours may best predict parental satisfaction with the relationship, we computed a ratio score. Both frequency scales showed good internal consistency in maternal report for their 2‐ to 6‐year‐old children (CRDQ: Cronbach's α = 0.85–0.88 in Briegel et al. 2019; current study: Cronbach's α = 0.89; CRC: Cronbach's α = 0.84–0.88 in Briegel et al. (2019); current study: Cronbach's α = 0.87).

#### 2.3.3 Dyadic Behaviour: Coding Interactive Behaviour (CIB) System

Behavioural observation was conducted with all families independent of child age. Mother–child interactions were analysed with the CIB (Feldman 1998), a global rating system with three scale levels (parent, child and dyad), each comprising composites (parent: sensitivity, intrusiveness, limit‐setting; child: involvement, withdrawal, compliance; dyad: reciprocity, negative states). Each composite is rated on multiple codes using a 5‐point Likert scale (1, *Minimum level of specific behaviour* to 5, *Maximum level of specific behaviour*), and a mean score is calculated per composite. We used the sensitivity composite (Cronbach's α = 0.86), an adapted intrusiveness composite (in line with recommendations of the Feldman's laboratory team, we excluded two codes with low internal consistency; Cronbach's α = 0.66–0.69), withdrawal (Cronbach's α = 0.77–0.78), involvement (Cronbach's α = 0.75–0.78), reciprocity (Cronbach's α = 0.91) and negative states (Cronbach's α= 0.66–0.73). Due to low internal consistencies, we refrained from analysing limit‐setting and compliance. Additional information on each composite is provided in the Supporting Information. Each task (free‐play task: 10 min, structured task: 8 min) was coded separately by research assistants. Coders were blind to the status of maternal mental disorder and time point. Following R. Feldman's lab guidelines, coders had to reach at least 85% agreement with master codings. Initial reliability across 18 videos ranged from 85% to 88% (roughly 20% of average measure ICC < 0.75). Reliability was reassessed with 28 videos, showing 76%–100% agreement (roughly 20% of average measure ICC < 0.75).

### 2.4 Statistical Analysis

Participants with missing behavioural observations were excluded from the respective analysis, following the recommendations provided in the respective manuals. All analyses were computed in R studio, version 2025.5.0.496 (Posit team 2025); code was refined using artificial intelligence (Bielefelder KI‐Interface 2023). Multilevel analyses were conducted to take account of the nested data structure (i.e., mother and child variables and two tasks nested in the dyad). For the questionnaires, we performed two‐way independent ANOVAs. For each model, assumptions were tested before we tested the effect of group on the respective outcome (with two contrasts: (1) both clinical groups (BPD and ad/D) vs. nonclinical group (CON); (2) BPD vs. ad/D group). Maternal education was entered as a second predictor, dichotomized into (a) a higher educational qualification (i.e., a qualification from secondary school or higher) or (b) a lower educational qualification (i.e., any qualification below this level). We subsequently added predictors to the CIB models and checked whether they improved the overall model fit (reports on each model, the model fits and the assumption tests are displayed in the Supporting Information). As we ran several statistical tests on the same research question, we applied the Bonferroni correction (self‐reported: *α* = 0.05/4 = 0.01; observational: *α* = 0.05/6 = 0.008).

## 3 Results

Descriptive statistics for the outcome variables are depicted in Table 2 (self‐reported outcomes) and in Table 3 (observed outcomes). Correlations between self‐reported and observed mother–child interactions are displayed in Table S2 and Table S3.

**TABLE 2:** Descriptive statistics of self‐reported dependent variables (PRQ and CRBI).

	BPD	ad/D	Comparison BPD vs. ad/D	CON	Comparison BPD and ad/D vs. CON
PRQ (maternal self‐report)					
*N*	114	47		59	
Attachment (mean/SD)	39.947 (12.425)	42.383 (12.498)	*p* = 0.127	49.475 (8.413)	***p* = 0.042**
Involvement (mean/SD)	44.860 (9.849)	46.404 (9.419)	*p* = 0.562, *d* = −0.16	51.966 (8.814)	***p* = 0.008, *d* = −0.70**
Relational Frustration (mean/SD)	63.395 (11.380)	56.872 (12.536)	***p* = 0.002, *d* = 0.56**	46.746 (8.444)	***p* < 0.001, *d* = 1.31**
CRBI (maternal self‐report)					
N	110	48		60	
Ratio (mean/SD)	2.111 (0.835)	2.364 (0.975)	*p* = 0.169, *d* = −0.29	2.829 (1.137)	***p* = 0.001, *d* = −0.67**

*Note:* PRQ values are displayed as *T*‐scores. Potential range of CRBI ratio scores: 0.143–7 (higher scores indicate a more positive perception of child's contribution to relationship). BPD: mothers with borderline personality disorder, AD/D: mothers with an anxiety disorder, depression or both, CON: mothers without any mental disorder.

**TABLE 3:** Descriptive statistics of observed dependent variables (CIB).

	Free‐play task			Structured play task (puzzle/feeding)		
	BPD	AD/D	CON	BPD	AD/D	CON
*N*	154	63	92	156	62	92
Mothers						
Sensitivity (mean/SD)	3.545 (0.593)	3.614 (0.464)	3.649 (0.459)	3.264 (0.638)	3.371 (0.547)	3.574 (0.464)
Intrusiveness (mean/SD)	1.368 (0.419)	1.289 (0.273)	1.300 (0.314)	1.444 (0.453)	1.365 (0.326)	1.350 (0.350)
Children						
Involvement (mean/SD)	3.741 (0.674)	3.646 (0.563)	3.716 (0.562)	3.164 (0.547)	3.266 (0.575)	3.410 (0.524)
Withdrawal (mean/SD)	1.328 (0.501)	1.369 (0.607)	1.316 (0.499)	1.600 (0.674)	1.479 (0.587)	1.485 (0.602)
Dyad						
Reciprocity (mean/SD)	3.734 (0.853)	3.773 (0.770)	3.873 (0.697)	3.338 (0.894)	3.541 (0.714)	3.673 (0.799)
Negative states (mean/SD)	1.627 (0.792)	1.540 (0.656)	1.507 (0.634)	1.905 (0.890)	1.738 (0.772)	1.614 (0.715)

*Note:* Potential range of CIB scores: 1–5 (higher scores indicate more display of respective dyadic behaviour). BPD: mothers with borderline personality disorder, AD/D: mothers with an anxiety disorder, depression or both, CON: mothers without any mental disorder.

### 3.1 Perception of Parenting and the Mother–Child Relationship: Self‐Report

Groups differed significantly in Relational Frustration (*F*(2, 214) = 20.964, *p* < 0.001). The a priori planned contrasts showed more relational frustration in the clinical groups (BPD and ad/D; M_CLIN_ = 61.50, SD_CLIN_ = 12.10) versus the nonclinical group (M_CON_ = 46.75, SD_CON_ = 8.44), *t*(214) = −5.04, *p* < 0.001, *d* = 1.31. Mothers with BPD (M_BPD_ = 63.40, SD_BPD_ = 11.38) were also more frustrated than mothers with ad/D (M_AD/D_ = 56.87, SD_AD/D_ = 12.54, see Table 2) (*t*(214) = −3.152, *p* = 0.002, *d* = 0.56). The main effects of education and the interaction effects were not significant in self‐reported data (see Table S25 for detailed information). Following the application of the Bonferroni correction (*α* = 0.01), Involvement fell just short of significance (*F*(2, 214) = 4.192, *p* = 0.016). Due to variance heterogeneity in the Attachment scale, a robust ANOVA using M‐measures of the median and bootstrapping was computed. Again, group membership significantly predicted differences in perceived attachment (*p* < 0.001). The post hoc test revealed that this effect was driven by differences between the clinical groups and the control group ($\hat{\Psi }$ = −14.348, *p* = 0.042; M_CLIN_ = 40.70, SD_CLIN_ = 12.50 vs. M_CON_ = 49.50, SD_CON_ = 8.41); no differences were identified between the BPD and the ad/D group (*p* = 0.127).

In the CRBI, mothers with BPD reported the lowest mean ratio (*T* = 39), followed by those with ad/D (*T* = 41). Mothers without any mental disorder reported the highest mean ratio (*T* = 45; see Table 2). The two‐way ANOVA revealed a significant main effect of group (*F*(2, 212) = 7.672, *p* = 0.001). The a priori defined contrasts showed significant differences between the clinical groups and the control group (*t*(212) = 3.391, *p* = 0.001, *d* = −0.67; M_CLIN_ = 2.19, SD_CLIN_ = 0.88 vs. M_CON_ = 2.83, SD_CON_ = 1.14), but not between the two clinical groups (*p* = 0.169).

### 3.2 Dyadic Behaviour in Mother–Child Interactions: Behavioural Observation

On a descriptive level, we observed low base rates for maternal intrusiveness and children's withdrawal, as well as for negative dyadic states, in both the free‐play and structured play tasks, and for children's involvement in the structured play task (see Table 3). Clinical (BPD, ad/D) and nonclinical (CON) group membership initially significantly predicted maternal sensitivity (Tables S4–S5) and dyadic reciprocity (Tables S14–S15), but this effect vanished once we included maternal education. When controlling for maternal education, mental disorder group membership did not significantly predict dyadic behaviour (see Table 4). We did not observe any significant effects of group membership on maternal intrusiveness (Tables S6–S9), child involvement (Tables S10–S11), child withdrawal (Tables S12–S13) or dyadic negative states (Tables S16–S17). The most consistent association of observed behaviour with a predictor was found for the context (type of task: free‐play vs. structured play) followed by education. We observed more sensitivity, less intrusiveness, more involvement, less withdrawal, more reciprocity and less negative states in the free‐play than the structured play task across groups. Education was associated with observed maternal behaviour but not child behaviour (see Table 4). Mothers with a lower educational background interacted less sensitive and more intrusive, and their dyadic interaction was less reciprocal and more characterized by negative states.

**TABLE 4:** Results of the multilevel models for the observed dyadic behaviour (CIB) with group, task and education as predictors.

		BPD and AD/D vs. CON	BPD vs. ad/D	Education low vs. high	Free‐play vs. structured	*R* ^2^
Mothers						
Sensitivity	*b* (SE), *p*	0.036 (0.020), *p* = 0.071	0.018 (0.034), *p* = 0.605	**0.091 (0.029), *p* = 0.002** **	**−0.210 (0.038), *p* < 0.001** ***	0.329
	Mean (SD)	*mean*_CLIN_ = 3.43 (0.60) *mean*_CON_ = 3.61 (0.46)	*mean*_BPD_ = 3.40 (0.63) *mean*_AD/D_ = 3.49 (0.52)	*mean*_low_ = 3.34 (0.66) *mean*_high_ = 3.56 (0.50)	*mean*_free_ = 3.59 (0.53) *mean*_struc_ = 3.38 (0.59)	
Intrusiveness	*b* (SE), *p*	−0.004 (0.017), *p* = 0.839	−0.028 (0.030), *p* = 0.356	**−0.095 (0.025), *p* < 0.001** ***	**0.091 (0.030), *p* = 0.003** **	0.386
	Mean (SD)	*mean*_CLIN_ = 1.42 (0.51) *mean*_CON_ = 1.33 (0.40)	*mean*_BPD_ = 1.46 (0.55) *mean*_ad/D_ = 1.35 (0.37)	*mean*_low_ = 1.53 (0.61) *mean*_high_ = 1.32 (0.37)	*mean*_free_ = 1.35 (0.46) *mean*_struc_ = 1.44 (0.49)	
Children						
Involvement	*b* (SE), *p*	0.031 (0.020), *p* = 0.133	−0.005 (0.035), *p* = 0.875	0.026 (0.030), *p* = 0.381	**−0.456 (0.043), *p* < 0.001** ***	0.300
	Mean (SD)	*mean*_CLIN_ = 3.45 (0.66) *mean*_CON_ = 3.56 (0.56)	*mean*_BPD_ = 3.45 (0.68) *mean*_ad/D_ = 3.46 (0.60)	*mean*_low_ = 3.43 (0.70) *mean*_high_ = 3.51 (0.59)	*mean*_free_ = 3.71 (0.62) *mean*_struc_ = 3.26 (0.56)	
Withdrawal	*b* (SE), *p*	−0.010 (0.021), *p* = 0.647	−0.014 (0.036), *p* = 0.703	−0.024 (0.031), *p* = 0.428	**0.207 (0.040), *p* < 0.001** ***	0.311
	Mean (SD)	*mean*_CLIN_ = 1.45 (0.61) *mean*_CON_ = 1.40 (0.56)	*mean*_BPD_ = 1.46 (0.61) *mean*_ad/D_ = 1.42 (0.60)	*mean*_low_ = 1.48 (0.62) *mean*_high_ = 1.41 (0.58)	*mean*_free_ = 1.33 (0.52) *mean*_struc_ = 1.54 (0.64)	
*DYAD*						
Reciprocity	*b* (SE), *p*	0.032 (0.029), *p* = 0.270	0.023 (0.050), *p* = 0.639	**0.130 (0.043), *p* = 0.002** **	**−0.302 (0.055), *p* < 0.001** ***	0.331
	Mean (SD)	*mean*_CLIN_ = 3.57 (0.86) *mean*_CON_ = 3.77 (0.76)	*mean*_BPD_ = 3.53 (0.90) *mean*_ad/D_ = 3.66 (0.75)	*mean*_low_ = 3.43 (0.94) *mean*_high_ = 3.74 (0.75)	*mean*_free_ = 3.78 (0.79) *mean*_struc_ = 3.48 (0.84)	
Negative states	*b* (SE), *p*	−0.023 (0.027), *p* = 0.381	−0.029 (0.046), *p* = 0.526	**−0.117 (0.039), *p* = 0.003** **	**0.210 (0.055), *p* < 0.001** ***	0.228
	Mean (SD)	*mean*_CLIN_ = 1.73 (0.82) *mean*_CON_ = 1.56 (0.68)	*mean*_BPD_ = 1.77 (0.85) *mean*_ad/D_ = 1.64 (0.72)	*mean*_low_ = 1.86 (0.92) *mean*_high_ = 1.58 (0.67)	*mean*_free_ = 1.57 (0.72) *mean*_struc_ = 1.79 (0.83)	

Abbreviations: BPD: mothers with borderline personality disorder, ad/D: mothers with an anxiety disorder, depression or both, CON: mothers without any mental disorder, CLIN: mothers with BPD and ad/D combined, Free: free‐play task, Struc: structured play task. For means of BPD vs. ad/D see also Table 3.

* *p* < 0.05.

** *p* < 0.01.

*** *p* < 0.001.

In addition to the intended analyses, we explored whether child age (dichotomized below/above 36 months) was a significant predictor of the observed behaviour. As the task was different for younger age groups, we wanted to ensure that any differences were not due to age. We compared children under 36 months, who engaged in a feeding situation with their mothers for the structured task, with and an older group (children 36 months and older, who solved a puzzle with their mothers for the structured task; see Table S18 for descriptives). Child age was a significant predictor of the observed behaviour, with the exception of sensitivity (Table S19, *p* = 0.135). Mothers with younger children were more intrusive (Table S20, *p* < 0.001), younger children were less involved (Table S21, *p* < 0.001) and more withdrawn (Table S22, *p* < 0.001), and dyads with younger children exhibited less reciprocity (Table S23, *p* = 0.002) and more negative states (Table S24, *p* = 0.037) during interaction.

## 4 Discussion

To our knowledge, this is the first study to comprehensively examine relational aspects of parenting and the parent–child relationship in mothers with BPD, compared to clinical (ad/D) and nonclinical (CON) control groups. In general, we observed significant differences between control (CON) and both clinical groups (BPD and ad/D combined) on relationship dimensions: Mothers with a mental disorder reported to be less attached, more relationally frustrated and perceived their child to invest less positive and more negative relationship behaviour than mothers from the control group. These results were not supported by behavioural observation data, where we neither detected statistically significant differences between clinical and nonclinical groups nor between both clinical groups. Therefore, families of all three groups interacted somewhat similarly in the behavioural observation tasks, while differences were more evident in the self‐reported dimensions of relationships. It should be noted that all mothers in the BPD group either had received or were currently receiving BPD‐specific treatment. Additionally, as will be discussed later, the clinical control group comprised mothers with disorders that are usually related to differential parenting styles (with depressive disorders being more strongly linked to disengagement and reduced sensitivity, whereas anxiety disorders are associated with greater intrusiveness) (Dib et al. 2019).

### 4.1 Mother–Child Relationship: Self‐Report Versus Observable Behaviour

We found only small to medium correlations between self‐reported and observed parenting, while the majority of our variables were not significantly correlated. The concordance was highest for the PRQ Attachment scale (with more perceived attachment corresponding to less observed intrusive behaviour and less observed negative states in the free‐play task as well as more observed sensitivity, more observed child's involvement and more observed reciprocity in the structured play task) and the CRBI ratio (with a higher proportion of perceived positive child contributions to the relationship corresponding to more observed sensitivity, more observed child's involvement, more observed reciprocity and less observed negative states in the structured play task) with the CIB composites. The observed behaviour in the structured play task correlated more frequently with self‐reported parenting than did the observations in the free‐play task. As the CRBI questionnaire reflects behaviour most closely, it is intuitive that the CRBI ratio would correlate more frequently with observed behaviour. The highest correlation between self‐reported and observed behaviour was indeed between the CRBI ratio and the CIB child involvement composite in the structured play task. Apparently, the perception of own parenting responds more closely to behaviour in structured task.

When comparing self‐reported and observed relationship behaviour, it was striking that the three groups did not differ from each other in terms of behavioural observation. This contradicts the majority of study results, which indicate differences in parenting among the examined disorders (e.g., Bernard et al. 2018; Hobson et al. 2009; Nicol‐Harper et al. 2007). Our results suggest that, in a controlled laboratory situation, mothers with various mental disorders are able to foster positive relationships with their children, as evidenced by low base rates of intrusiveness and negative states. This may correspond to an observer effect (Kälvemark Sporrong et al. 2022), which means that these mothers have an understanding of how they should behave and that they can maintain this behaviour for short observation periods. Questionnaires, in contrast, may tend to measure enduring, context‐independent competencies and mental summaries. A possible assumption may be that mothers with mental disorders are unable to maintain these behaviours permanently. Qualitative research on parents with BPD revealed that, while they can be highly reflective at one time, they can also lack insight into how their children experience their relationship and parenting behaviours the next (Dunn et al. 2020). It should also be noted that, in the BPD group in particular, only mothers who were either currently or previously in therapy participated in our study. Nevertheless, mothers in the ad/D group attended outpatient psychotherapy at a similar rate to those in the BPD group, although the former were less often treated in an inpatient setting. Therefore, our clinical comparison group is similar to the BPD group in that it is a group that has already received treatment. Differences in the BPD group may therefore also be attributable to the effects of therapy that may have already begun to take hold. Finally, the free‐play task may generate less dysfunctional parenting behaviour than tasks that explicitly demand parenting skills for successful completion. The discrepancy between self‐reported and observed aspects of the mother–child relationship may also be due to negative self‐report bias among mothers with BPD. These mothers have a less positive perception of their competencies than mothers without personality disorders (Ramsauer et al. 2016) and report more guilt and despair in relation to parenting (Dunn et al. 2020). As Rosenbach et al. (2025, manuscript, submitted for publication) demonstrated, the mothers in our sample reported higher levels of parenting distress and lower levels of parental satisfaction than mothers without mental disorders.

### 4.2 Contextual and Child Influences on Observable Behaviour

Structured tasks are assumed to be experienced as more stressful and have been found to elicit more variation in behaviours—especially more negative dyadic behaviour—than free‐play situations (Blacher et al. 2013), hence ‘revealing more meaningful behaviors’ (Krijnen et al. 2023, 11). Consistent with this, we assumed that negative interaction patterns would be more prevalent across all three groups in the structured task than in the free‐play context. In fact, we found clear evidence supporting this assumption: Across all groups, mother–child interactions were less positive in the structured task. Results of previous studies imply that mothers without mental disorders experience more stress during structured tasks, leading to more negative child affect and more tense interactions (Krijnen et al. 2023).

Additionally, our exploratory analyses revealed evidence of the influence of child characteristics: With the exception of sensitivity, less favourable dyadic behaviour was observed in interactions between mothers and younger children. Although child age is often cited as a relevant factor in parent–child interactions (Bornstein 2016; Sanson et al. 2018), the concrete effects of child age on mother–child interactions are rarely investigated, to our knowledge. The results of the NICHD study imply that the behaviour of parents and children is, at best, only moderately stable over time (NICHD Early Child Care Research Network 2003). This may suggest that children develop certain skills for building and maintaining relationships as they grow older. To illustrate, the capacity to exhibit prosocial behaviour, such as cooperation with others and engaging in reciprocal interactions, matures from the second year of life onwards (Brownell 2013). Depending on developmental processes in sociocognitive understanding, prosociality continues to increase as development progresses (Malti and Dys 2018). Although parent–child relationships are characterized by an imbalance of power, they are also reciprocal relationships in which the child plays an active role in shaping the relationship (Frosch et al. 2021; Heinrichs et al. 2020; Russell et al. 2002). Younger children, for instance, seem to test boundaries more and invest less in their relationship with their mothers. Consequently, mothers with younger children may be more inclined to set boundaries, which can sometimes also be reflected in higher intrusiveness. This assumption stems from interactions between parents and children with externalizing behaviours, where more harsh reactions are exhibited by parents to their children's noncompliance (Scaramella and Leve 2004). However, the specific effects of a child's age on observable behaviour are not yet clear and require further research.

### 4.3 Disorder‐Specific vs. Transdiagnostic Associations With Various Aspects of the Mother–Child Relationship

As expected, both clinical groups differed from the healthy group, which is consistent with the findings of earlier studies. Unexpectedly, we found no difference between the two clinical groups in perceived closeness or the general perception of the child's contribution to the relationship. It is noteworthy that mothers in both groups reported being involved with and attached to their children (albeit to a lesser extent than the CON mothers). This is in line with previous studies which, for example, have found that mothers with BPD have a strong affection for their children and care deeply for them (Eyden et al. 2016). Becoming a parent can enhance the well‐being of individuals with BPD, creating a sense of purpose and achievement and is also often the motivation behind seeking treatment (Kasiviswanathan et al. 2025). We propose that mothers with BPD, just like mothers with other mental disorders, view the relationship with their child positively (albeit less than CON mothers), enjoy it and also perceive their child's contributions to the relationship. At the same time, we suspect that this general ability is more quickly disrupted in BPD in challenging situations (e.g., perceived rejection by the child) and cannot be maintained continuously during such periods. This is supported by the fact that we found a significant difference between the two clinical groups in terms of perceived frustration in and with the relationship (PRQ Relational Frustration). The scale describes the mothers' tendency to overreact and become frustrated in common parenting situations, e.g., when their children are misbehaving (Kamphaus and Reynolds 2015). The emotional instability that occurs in BPD may be transferred to the relationship with the child (Florange and Herpertz 2019). In a qualitative study, parents with BPD traits reported that feelings such as anger, despair or stress can be triggered by their child's behaviour, resulting in intense emotional responses and difficulties in attuning to their child (Dunn et al. 2020; Kasiviswanathan et al. 2025). The parent–child relationship can also be characterized by enmeshment and parentification (Dunn et al. 2020; Kasiviswanathan et al. 2025). This can be accompanied by unrealistic or excessive demands and expectations of the child (cp. Wendland et al. 2014), leading to greater frustration when these expectations are not met—especially if they are during high arousal not able to reflect their own expectations.

Overall, our results support transdiagnostic associations between mental disorders and relationship dimensions rather than disorder‐specific associations for relational cognitions—with the exception of relational frustration. This contradicts the results for self‐reported parenting skills reflecting an educational focus, which were obtained from the same sample (Rosenbach et al. 2025, manuscript submitted for publication). However, it might be of interest to also consider heterogeneity within groups. Taking the attachment scale results from the PRQ as an example, both clinical groups displayed a significantly higher variability around the mean which required robust testing. Although the below‐average *T*‐scores suggest that both clinical groups could benefit from attachment‐related interventions, the considerable heterogeneity within each group highlights the need for a more nuanced approach. Identifying subgroups may help tailor interventions more effectively by addressing specific strengths and difficulties within the groups.

### 4.4 Limitations

The current study is limited in several ways: We used a mixed clinical control group comprising anxiety and depressive disorders, even though some studies imply that different mechanisms in parenting are operating in these two disorders. For example, mothers with anxiety disorders are assumed to exhibit more anxious misinterpretations and escalations of the mother–infant interaction (Petzoldt et al. 2016), while mothers with depression may be especially challenged by rumination processes (DeJong et al. 2016). Anxiety symptoms are sometimes also evident to be associated with more favourable dyadic behaviour such as synchrony (Lemus et al. 2022). However, since depression and anxiety disorders are highly comorbid (Jacobi et al. 2014), we decided to treat mothers with such disorders as one group. A second limitation is evident in the correlation between self‐reported and observed behaviour. While the questionnaires were only available to families with children aged 2 years and up, the behavioural observations also included families with younger children. Therefore, our correlational results only provide information on the subsample of families 2 years and up. Additionally, the behavioural observation took place in a laboratory setting. This setting may have resulted in less‐naturalistic observations of the interaction and may have caused mothers to behave differently in this brief, artificial situation than they would have at home (cp. Gardner 2000). Finally, the study focused more on the relationship dimensions of mothers than of children but children's characteristics, including how they perceive and think about the relationship, can also influence their mothers' parenting (Taraban and Shaw 2018). It should also be considered that, at times, the reliability of our coders was below the level of moderate agreement.

The present study is also characterized by several strengths: large sample sizes in all three groups, clear group membership as reflected by results of structured interviews, blind coding of behaviour, using both self‐report and observational methods, and introducing a clinical control group.

### 4.5 Implications

Mothers with BPD are often assumed to exhibit highly dysfunctional parenting behaviour. However, this study demonstrates that mothers with BPD, like mothers with other mental disorders, can behave positively and sensitively towards their children. Overall, the short interactions were not characterized by dysfunctionality, even during more stressful tasks. We assume that mothers with mental disorders are not always able to maintain these skills, as reflected in the more context‐independent questionnaires. We suggest two approaches to support mothers with a mental disorder: (1) Interventions should address potentially distorted perceptions of parenting competencies (Dunn et al. 2020) as well as negative perceptions of the child's (mis)behaviour or dissatisfaction with the relationship. A hostile and dismissive interpretation of children's behaviour is a common predictor of child abuse and neglect (Azar et al. 2012). (2) Clinicians may help mothers to maintain appropriate parenting skills over longer periods of time and in various challenging situations. Situations in which children misbehave or display strong emotions are particularly challenging for affected mothers, and higher BPD symptoms are associated with greater hostility, blame and misattributions (Dáu and Milan 2022; Kiel et al. 2017). Interventions should therefore concentrate on teaching mothers how to deal with stressful situations and resolve conflicts (Florange and Herpertz 2019; Renneberg and Rosenbach 2016). Our results indicate that mothers with BPD face comparable challenges to those with anxiety disorders or depression, suggesting that the same intervention components can be utilized. At the same time, the findings from the Attachment scale point to meaningful variability within both clinical groups, underlining the importance of identifying individual profiles of strengths and difficulties to better tailor intervention strategies.

For researchers, we suggest moving to (1) further investigation of behavioural observations for longer time periods (as to explore how long positive skills can be maintained) and (2) what may be the key drivers causing parenting deficits in parenting. While groups may not differ in result patterns, mechanisms that cause the observable patterns may still differ. This may also include behavioural observations of not only the dyad but also the family. For example, having multiple children consistently predicted that the mothers in our sample would engage in more self‐reported maladaptive parenting skills, i.e., overactivity (Rosenbach et al. 2025, manuscript submitted for publication). For this reason, it would be beneficial to conduct more ecologically valid observations (e.g., home visits where siblings and competing demands are also present or observations over a longer period of time to mitigate observer effects).

## 5 Conclusion

This study makes a significant contribution to our understanding of the associations between maternal mental disorders and mother–child relationships. Overall, it shows that mothers with BPD experience similar challenges in the relationship with their children as mothers with anxiety disorders and depression. Both clinical groups demonstrated differences compared with mothers without mental disorders, although these differences were mostly evident in self‐report and not in observed interactions. This suggests that while mothers with mental disorders are capable of fostering positive, sensitive relationships with their children, they may not always be able to maintain these relationships and may have more negative self‐views. Research may move forward by focusing on more ecologically valid observations and focus also on mechanisms instead of observable behaviour patterns only. Clinicians may focus on providing parenting skills that bolster skills that help in dealing with conflicts in a surrounding of multiple demands.

## Author Contributions

Conceptualization, A.J. and N.H.; methodology, A.J.; formal analysis, A.J. (using the Bielefelder KI Interface, an artificial intelligence tool, to search for solutions to problems occurring within the R script and to refine code); investigation, A.J.; writing – original draft preparation, A.J. with the support of NH; writing – review and editing, N.H., B.R., R.K. and S.S.; visualization, A.J.; supervision, N.H. All authors have read and agreed to the final version of the manuscript.

## Funding

This study is based on data from a research project investigating family climate in families with mothers with mental disorders which was funded by the Federal Ministry of Education and Research (funding code: 01KR1805C). The first author (A.J.) was partially employed in this research project, and several other co‐authors (N.H., B.R., S.S. and R.K.) were involved as Principal Investigators.

## Ethics Statement

The procedure for the study was reviewed by the Ethics Committee of the German Association of Psychology (Deutsche Gesellschaft für Psychologie, DGPs; protocol code RennebergBabette2019‐07‐29VADM, accepted 26 September 2019). Written informed consent was obtained from all subjects involved in the study.

## Conflicts of Interest

Babette Renneberg is a co‐author of the group training ‘Borderline and motherhood’ that is being evaluated in the ‘ProChild’ project.

## Supporting information




Table S1:
**Table S2:** Correlations between self‐reported (PRQ, CRBI) and observed mother–child interaction (CIB) in the free‐play task.
**Table S3:** Correlations between self‐reported (PRQ, CRBI) and observed mother–child interaction (CIB) in the structured play task.
**Table S4:** Modelling of parental sensitivity.
**Table S5:** Model fit of parental sensitivity.
**Table S6:** Modelling of parental intrusiveness (revised composite after excluding parental anxiety and parental depressed mood).
**Table S7:** Model fit of parental intrusiveness (revised).
**Table S8:** Modelling of parental intrusiveness (original composite with parental anxiety and parental depressed mood).
**Table S9:** Model fit of parental intrusiveness (original).
**Table S10:** Modelling of children's involvement.
**Table S11:** Model fit of children's involvement.
**Table S12:** Modelling of children's withdrawal.
**Table S13:** Model fit of children's withdrawal.
**Table S14:** Modelling of dyadic reciprocity.
**Table S15:** Model fit of dyadic reciprocity.
**Table S16:** Modelling of dyadic negative states.
**Table S17:** Model fit of dyadic negative states.
**Table S18:** Observed maternal, child, and dyadic behaviour (CIB) per group, child age, and task.
**Table S19:** Modelling of parental sensitivity, including dichotomized child age as a predictor.
**Table S20:** Modelling of parental intrusiveness, including dichotomized child age as a predictor.
**Table S21:** Modelling of child involvement, including dichotomized child age as a predictor.
**Table S22:** Modelling of child withdrawal, including dichotomized child age as a predictor.
**Table S23:** Modelling of dyadic reciprocity, including dichotomized child age as a predictor.
**Table S24:** Modelling of dyadic negative states, including dichotomized child age as a predictor.
**Table S25:** Results of two‐way independent ANOVAs with group and education as predictors.

## Data Availability

The scripts used for analysing the present data are available in the OSF repository, https://doi.org/10.17605/OSF.IO/DXNGA. Raw data supporting the conclusions of this article will be uploaded in the repository in completely anonymized form after the publication of all preregistered research questions of the consortium. Until then, the datasets used and/or analysed during the current study are available from the corresponding author on reasonable request.
