# Supplementary material for: Mother–Child Relationship Quality in the Presence of Maternal Mental Disorders: Do Self‐Report and Behavioural Observation Differ?
Source: Clin Psychol Psychother. 2026 Mar 7;33(2):e70227. doi: 10.1002/cpp.70227 (PMC12966951; doi:10.1002/cpp.70227)
Supplement: Supplementary file 1 — Table S1: Table S2: Correlations between self‐reported (PRQ, CRBI) and observed mother–child interaction (CIB) in the free‐play task. Table S3: Correlations between self‐reported (PRQ, CRBI) and observed mother–child interaction (CIB) in the structured play task. Table S4: Modelling of parental sensitivity. Table S5: Model fit of parental sensitivity. Table S6: Modelling of parental intrusiveness (revised composite after excluding parental anxiety and parental depressed mood). Table S7: Model fit of parental intrusiveness (revised). Table S8: Modelling of parental intrusiveness (original composite with parental anxiety and parental depressed mood). Table S9: Model fit of parental intrusiveness (original). Table S10: Modelling of children's involvement. Table S11: Model fit of children's involvement. Table S12: Modelling of children's withdrawal. Table S13: Model fit of children's withdrawal. Table S14: Modelling of dyadic reciprocity. Table S15: Model fit of dyadic reciprocity. Table S16: Modelling of dyadic negative states. Table S17: Model fit of dyadic negative states. Table S18: Observed maternal, child, and dyadic behaviour (CIB) per group, child age, and task. Table S19: Modelling of parental sensitivity, including dichotomized child age as a predictor. Table S20: Modelling of parental intrusiveness, including dichotomized child age as a predictor. Table S21: Modelling of child involvement, including dichotomized child age as a predictor. Table S22: Modelling of child withdrawal, including dichotomized child age as a predictor. Table S23: Modelling of dyadic reciprocity, including dichotomized child age as a predictor. Table S24: Modelling of dyadic negative states, including dichotomized child age as a predictor. Table S25: Results of two‐way independent ANOVAs with group and education as predictors. [file CPP-33-e70227-s001.docx]

**Mother-Child Relationship Quality in the Presence of Maternal Mental Disorders: Do Self-Report and Behavioral Observation Differ? – Supplementary Materials**

## Information regarding the PRQ and CRBI scales.

We used three scales of the PRQ, *Attachment, Involvement,* and *Relational Frustration. Attachment* reflects feelings of closeness and understanding towards the child (e.g., “I enjoy spending time with my child”), while *Involvement* captures the parent’s knowledge about their child’s activities and the number of joint activities (e.g., “My child and I play games together”). *Relational Frustration* describes the parental perception of frustration in relation to the child (e.g., “I feel overwhelmed being a parent”).

For the PRQ, a maximum of two missing cases per scale was appropriate and missing values were imputed using the most frequently selected response for that scale as described in the manual (Kamphaus & Reynolds, 2015). When more missing values occurred within one scale, the scale was omitted for the respective participant.

Some PRQ subscales were not included in the analyses. For the PRQ, this was the *Discipline Practices* scale, which stood out due to its extremely low T-values. These values were deemed implausible and the scale was therefore excluded from further analysis to avoid misinterpretation. Additionally, the two PRQ scales, *Communication* and *Satisfaction with School*, were omitted as they were only used by mothers with older children, who were underrepresented in the present sample, resulting in insufficient statistical power for an additional analysis. The PRQ *Parenting Confidenc*e scale was not reported to avoid overlap with subproject 1. Information regarding the excluded subscales is reported in Table S1.

Table S1. Additional descriptives for the PRQ.

|  | BPD | ADD | CON |
| --- | --- | --- | --- |
| PRQ: Discipline Practices (mean/SD) | 30.632  (7.988) | 29.638  (7.623) | 27.153  (6.565) |
| PRQ: Parenting Confidence (mean/SD) | 30.877  (11.893) | 36.468  (12.190) | 50.136 (10.083) |
| PRQ: Communication (mean/SD)^1^ | 38.263  (11.165) | 47.167  (2.563) | 47.500  (11.726) |
| PRQ: Satisfaction With School (mean/SD)^1^ | 44.500  (11.673) | 44.667  (7.916) | 54.167  (8.085) |
| *Note.* PRQ values are displayed as T values. BPD: mothers with borderline personality disorder, AD/D: mothers with an anxiety disorder, depression, or both, CON: mothers without any mental disorder. ^1^ information available from 31 mothers (n_BPD_ = 19, n_ADD_ = 6, n_CON_ = 6). | | | |

For the CRBI, a maximum of two missing values were deemed appropriate. Missing values were imputed using the grand mean (cp., Briegel et al., 2019 and private discussion with W. Briegel in 07/2025).

## Information regarding the CIB scales.

*Maternal sensitivity* consisted of the following codes: acknowledging, imitating, elaborating, parent joint attention, parent positive affect, vocal appropriateness, range of affect, resourcefulness, praising, affectionate touch, parent supportive presence, and enthusiasm. *Maternal intrusiveness* consisted of the following codes: forcing, overriding, anger, hostility, and criticizing (due to low internal consistency and recommendations of the Feldman’s laboratory team, we excluded parental anxiety and depressive affect). *Child’s withdrawal* consisted of the following codes: negative emotionality, withdrawal, emotional lability, and child avoidance of the parent. *Child’s involvement* consisted of the following codes: child joint attention, child positive affect, child affection to parent, alert, fatigue (revised), vocalization, initiation, competent use of environment, and creative symbolic play. *Dyadic reciprocity* consisted of the following codes: reciprocity, adaptation-regulation, and fluency. *Dyadic negative states* consisted of the following codes: constriction and tension.

## Correlations between self-reported and observed mother-child interactions.

Table S2. Correlations between self-reported (PRQ, CRBI) and observed mother-child interaction (CIB) in the free-play task.

|  | CIB Sensitivity | CIB Intrusiveness | CIB Involvement | CIB Withdrawal | CIB Reciprocity | CIB Neg. States |
| --- | --- | --- | --- | --- | --- | --- |
| PRQ Attachment | .13 | **-.22**** | -.01 | .01 | .09 | **-.15*** |
|  | [-.00, .27] | **[-.34, -.08]** | [-.14, .13] | [-.13, .14] | [-.05, .22] | **[-.28, -.02]** |
| PRQ Involvement | .13 | -.09 | -.02 | .01 | .07 | -.13 |
|  | [-.01, .26] | [-.22, .05] | [-.15, .12] | [-.13, .14] | [-.06, .21] | [-.26, .01] |
| PRQ Relational Frustration | -.09 | .07 | .01 | -.03 | -.07 | .11 |
|  | [-.22, .05] | [-.07, .20] | [-.12, .15] | [-.16, .11] | [-.21, .06] | [-.02, .25] |
| CRBI Ratio | .07 | -.06 | -.07 | .00 | .01 | -.05 |
|  | [-.07, .20] | [-.20, .07] | [-.21, .06] | [-.13, .14] | [-.12, .15] | [-.19, .09] |
| *Note.* *N* = 204. Values in square brackets indicate the 95% confidence interval for each correlation. The confidence interval is a plausible range of population correlations that could have caused the sample correlation (Cumming, 2014). To integrate the correlations between self-report and observation, we averaged (z-transformed and back-transformed) the correlations. Negatively polarized scales (relational frustration, intrusiveness, withdrawal and negative states) were reverse-coded in advance, meaning that higher values consistently represented a more functional relationship. The resulting mean correlation was $\bar{r}$ = .06. * indicates *p* < .05. ** indicates *p* < .01 | | | | | | |

Table S3. Correlations between self-reported (PRQ, CRBI) and observed mother-child interaction (CIB) in the structured play task.

|  | CIB Sensitivity | CIB Intrusiveness | CIB Involvement | CIB Withdrawal | CIB Reciprocity | CIB Neg. States |
| --- | --- | --- | --- | --- | --- | --- |
| PRQ Attachment | **.20**** | -.11 | **.24**** | .00 | **.18**** | -.11 |
|  | **[.06, .32]** | [-.24, .03] | **[.10, .36]** | [-.13, .14] | **[.04, .31]** | [-.24, .03] |
| PRQ Involvement | **.21**** | **-.14*** | **.22**** | -.02 | .12 | -.10 |
|  | **[.08, .34]** | **[-.27, -.01]** | **[.09, .34]** | [-.16, .11] | [-.02, .25] | [-.23, .04] |
| PRQ Relational Frustration | -.13 | .07 | **-.15*** | -.04 | -.07 | .07 |
|  | [-.26, .01] | [-.07, .20] | **[-.28, -.02]** | [-.17, .10] | [-.20, .07] | [-.07, .20] |
| CRBI Ratio | **.26**** | -.12 | **.29**** | -.12 | **.23**** | **-.20**** |
|  | **[.13, .38]** | [-.25, .01] | **[.16, .41]** | [-.25, .02] | **[.10, .36]** | **[-.33, -.07]** |
| *Note.* *N* = 205. Values in square brackets indicate the 95% confidence interval for each correlation. The confidence interval is a plausible range of population correlations that could have caused the sample correlation (Cumming, 2014). To integrate the correlations between self-report and observation, we averaged (z-transformed and back-transformed) the correlations. Negatively polarized scales (relational frustration, intrusiveness, withdrawal and negative states) were reverse-coded in advance, meaning that higher values consistently represented a more functional relationship. The resulting mean correlation was $\bar{r}$ = .13. * indicates *p* < .05. ** indicates *p* < .01. | | | | | | |

## Multilevel modelling of sensitivity (CIB)

The assumptions were tested using VIF and tolerance scores to check for multicollinearity (all VIFs were less than 10 and tolerances were greater than 0.01), the Durbin-Watson test to check for dependent residuals (the Durbin-Watson statistic was non-significant, i.e. between 1 and 3) and QQ plots to check for homoscedasticity (visual inspection revealed no issues). We used the lme4 package (Bates et al., 2015) to perform the multilevel analysis.

Table S4. Modelling of parental sensitivity.

|  | Model 1 | Model 2 | Model 3 | Model 4 | Model 5 | Model 6 | |
| --- | --- | --- | --- | --- | --- | --- | --- |
| Intercept | 3.482, *p* < .001*** | 3.502, *p* < .001*** | 3.607, *p* < .001*** | 3.570, *p* < .001*** | 3.603, *p* < .001*** | 3.598, *p* < .001*** | |
|  | (0.026) | (0.027) | (0.033) | (0.035) | (0.041) | (0.041) | |
| BPD/ADD vs. CON |  | **0.055, *p* = .004**** | **0.055, *p* = .004**** | 0.036, *p* = .071+ | 0.036, *p* = .217 | 0.005, *p* = .874 | |
|  |  | **(0.019)** | **(0.019)** | (0.020) | (0.029) | (0.033) | |
| BPD vs. ADD |  | 0.045, *p* = .174 | 0.044, *p* = .182 | 0.018, *p* = .604 | 0.045, *p* = .213 | 0.037, *p* = -397 | |
|  |  | (0.033) | (0.033) | (0.034) | (0.036) | (0.044) | |
| Education low vs. high |  |  |  | **0.091, *p* = .002**** | 0.051, *p* = .155 | 0.051, *p* = .155 | |
|  |  |  |  | **(0.029)** | (0.036) | (0.036) | |
| BPD&ADD vs. CON * low vs. high |  |  |  |  | -0.001, *p* = .967 | -0.001, *p* = .968 | |
|  |  |  |  |  | (0.029) | (0.029) | |
| BPD vs. ADD * low vs. high |  |  |  |  | **-0.084, *p* = .021*** | **-0.084, *p* = .020*** | |
|  |  |  |  |  | **(0.036)** | **(0.036)** | |
| Free-play vs. structured |  |  | **-0.210, *p* < .001***** | **-0.210, *p* < .001***** | **-0.211, *p* < .001***** | **-0.199, *p* < .001***** | |
|  |  |  | **(0.038)** | **(0.038)** | **(0.038)** | **(0.040)** | |
| BPD&ADD vs. CON * free-play vs. structured |  |  |  |  |  | **0.062, *p* = .027*** | |
|  |  |  |  |  |  | **(0.028)** | |
| BPD vs. ADD * free-play vs. structured |  |  |  |  |  | 0.016, *p* = .753 | |
|  |  |  |  |  |  | (0.049) | |
| Num.Obs. | 619 | 619 | 619 | 619 | 619 | 619 | |
| R2 Marg. | 0.000 | 0.025 | 0.059 | 0.078 | 0.088 | 0.094 | |
| R2 Cond. | 0.259 | 0.263 | 0.329 | 0.329 | 0.332 | 0.341 | |
| AIC | 1049.9 | 1052.3 | 1029.2 | 1026.7 | 1035.1 | 1043.0 | |
| BIC | 1063.2 | 1074.5 | 1055.7 | 1057.7 | 1074.9 | 1091.7 | |
| ICC | 0.3 | 0.2 | 0.3 | 0.3 | 0.3 | 0.3 | |
| RMSE | 0.44 | 0.44 | 0.41 | 0.41 | 0.41 | 0.41 | |
| *Note.* + p < .10, * p < .05, ** p < .01, *** p < .001. Standard errors are displayed in brackets. | | | | | | |  |

Table S5. Model fit of parental sensitivity.

|  | Test statistic |
| --- | --- |
| Model 1 vs. Model 2 | *χ²*(2) = 12.72, ***p* = .002** |
| Model 2 vs. Model 3 | *χ²*(1) = 29.88, ***p* < .001** |
| Model 3 vs. Model 4 | *χ²*(1) = 9.84, ***p* = .002** |
| Model 4 vs. Model 5 | *χ²*(2) = 5.75, *p* = .056 |
| Model 5 vs. Model 6 | *χ²*(2) = 5.70, *p* = .058 |

## Multilevel modelling of intrusiveness (CIB).

The assumptions were tested using VIF and tolerance scores to check for multicollinearity (all VIFs were less than 10 and tolerances were greater than 0.01), the Durbin-Watson test to check for dependent residuals (the Durbin-Watson statistic was non-significant, i.e. between 1 and 3) and QQ plots to check for homoscedasticity (visual inspection revealed potential issues). The results should therefore be interpreted with caution. We used the lme4 package (Bates et al., 2015) to perform the multilevel analysis.

Table S6. Modelling of parental intrusiveness (revised composite after excluding parental anxiety and parental depressed mood).

|  | Model 1 | Model 2 | Model 3 | Model 4 | Model 5 | Model 6 | |
| --- | --- | --- | --- | --- | --- | --- | --- |
| Intercept | 1.397, *p* < .001*** | 1.378, *p* < .001*** | 1.332, *p* < .001*** | 1.371, *p* < .001*** | 1.347, *p* < .001*** | 1.343, *p* < .001*** | |
|  | (0.023) | (0.024) | (0.028) | (0.030) | (0.035) | (0.036) | |
| BPD/ADD vs. CON |  | -0.024, *p* = .163 | -0.024, *p* = .163 | -0.004, *p* = .839 | -0.004, *p* = .879 | -0.002, *p* = .943 | |
|  |  | (0.017) | (0.017) | (0.017) | (0.026) | (0.028) | |
| BPD vs. ADD |  | -0.056, *p* = .057+ | -0.056, *p* = .059+ | -0.028, *p* = .355 | -0.047, *p* = .138 | -0.061, *p* = .108 | |
|  |  | (0.029) | (0.029) | (0.030) | (0.032) | (0.038) | |
| Education low vs. high |  |  |  | **-0.095, *p* < .001***** | **-0.067, *p* = .037*** | **-0.067, *p* = .037*** | |
|  |  |  |  | **(0.025)** | **(0.032)** | **(0.032)** | |
| BPD&ADD vs. CON * low vs. high |  |  |  |  | 0.001, *p* = .978 | 0.001, *p* = .978 | |
|  |  |  |  |  | (0.026) | (0.026) | |
| BPD vs. ADD * low vs. high |  |  |  |  | 0.060, *p* = .059+ | 0.060, *p* = .058+ | |
|  |  |  |  |  | (0.032) | (0.032) | |
| Free-play vs. structured |  |  | **0.091, *p* = .003**** | **0.091, *p* = .003**** | **0.091, *p* = .003**** | **0.099, *p* = .003**** | |
|  |  |  | **(0.030)** | **(0.030)** | **(0.030)** | **(0.033)** | |
| BPD&ADD vs. CON * free-play vs. structured |  |  |  |  |  | -0.004, *p* = .864 | |
|  |  |  |  |  |  | (0.023) | |
| BPD vs. ADD * free-play vs. structured |  |  |  |  |  | 0.026, *p* = .512 | |
|  |  |  |  |  |  | (0.040) | |
| Num.Obs. | 619 | 619 | 619 | 619 | 619 | 619 | |
| R2 Marg. | 0.000 | 0.016 | 0.025 | 0.054 | 0.061 | 0.061 | |
| R2 Cond. | 0.365 | 0.368 | 0.384 | 0.386 | 0.389 | 0.387 | |
| AIC | 817.0 | 825.3 | 823.5 | 817.3 | 828.1 | 842.1 | |
| BIC | 830.3 | 847.4 | 850.1 | 848.3 | 868.0 | 890.8 | |
| ICC | 0.4 | 0.4 | 0.4 | 0.4 | 0.3 | 0.3 | |
| RMSE | 0.33 | 0.33 | 0.32 | 0.32 | 0.32 | 0.32 | |
| *Note.* + p < .10, * p < .05, ** p < .01, *** p < .001. Standard errors are displayed in brackets. | | | | | | |  |

Table S7. Model fit of parental intrusiveness (revised).

|  | Test statistic |
| --- | --- |
| Model 1 vs. Model 2 | *χ²*(2) = 7.34, ***p* = .026** |
| Model 2 vs. Model 3 | *χ²*(1) = 8.90, ***p* = .003** |
| Model 3 vs. Model 4 | *χ²*(1) = 13.81, ***p* < .001** |
| Model 4 vs. Model 5 | *χ²*(2) = 3.84, *p* = .147 |
| Model 5 vs. Model 6 | *χ²*(2) = 0.43, *p* = .805 |

Table S8. Modelling of parental intrusiveness (original composite with parental anxiety and parental depressed mood).

|  | Model 1 | Model 2 | Model 3 | Model 4 | Model 5 | Model 6 | |
| --- | --- | --- | --- | --- | --- | --- | --- |
| Intercept | 1.367, *p* < .001*** | 1.353, *p* < .001*** | 1.319, *p* < .001*** | 1.348, *p* < .001*** | 1.323, *p* < .001*** | 1.323, *p* < .001*** | |
|  | (0.018) | (0.019) | (0.023) | (0.024) | (0.028) | (0.029) | |
| BPD/ADD vs. CON |  | -0.014, *p* = .288 | -0.014, *p* = .287 | 0.001, *p* = .962 | -0.002, *p* = .933 | 0.003, *p* = .901 | |
|  |  | (0.013) | (0.013) | (0.014) | (0.020) | (0.023) | |
| BPD vs. ADD |  | -0.040, *p* = .089+ | -0.039, *p* = .092+ | -0.019, *p* = .433 | -0.037, *p* = .139 | -0.038, *p* = .208 | |
|  |  | (0.023) | (0.023) | (0.024) | (0.025) | (0.030) | |
| Education low vs. high |  |  |  | **-0.070, *p* < .001***** | -0.041, *p* = .101 | -0.041, *p* = .101 | |
|  |  |  |  | **(0.020)** | (0.025) | (0.025) | |
| BPD&ADD vs. CON * low vs. high |  |  |  |  | 0.003, *p* = .871 | 0.003, *p* = .872 | |
|  |  |  |  |  | (0.020) | (0.020) | |
| BPD vs. ADD * low vs. high |  |  |  |  | **0.058, *p* = .021*** | **0.058, *p* = .021*** | |
|  |  |  |  |  | **(0.025)** | **(0.025)** | |
| Free-play vs. structured |  |  | **0.068, *p* = .008**** | **0.068, *p* = .008**** | **0.068, *p* = .008**** | **0.068, *p* = .014*** | |
|  |  |  | **(0.025)** | **(0.025)** | **(0.025)** | **(0.027)** | |
| BPD&ADD vs. CON * free-play vs. structured |  |  |  |  |  | -0.009, *p* = .637 | |
|  |  |  |  |  |  | (0.019) | |
| BPD vs. ADD * free-play vs. structured |  |  |  |  |  | 0.002, *p* = .960 | |
|  |  |  |  |  |  | (0.034) | |
| Num.Obs. | 619 | 619 | 619 | 619 | 619 | 619 | |
| R2 Marg. | 0.000 | 0.011 | 0.019 | 0.043 | 0.054 | 0.055 | |
| R2 Cond. | 0.312 | 0.315 | 0.329 | 0.330 | 0.333 | 0.331 | |
| AIC | 556.4 | 567.7 | 568.2 | 564.2 | 573.9 | 588.7 | |
| BIC | 569.7 | 589.9 | 594.7 | 595.2 | 613.7 | 637.4 | |
| ICC | 0.3 | 0.3 | 0.3 | 0.3 | 0.3 | 0.3 | |
| RMSE | 0.28 | 0.28 | 0.28 | 0.28 | 0.28 | 0.28 | |
| *Note.* + p < .10, * p < .05, ** p < .01, *** p < .001. Standard errors are displayed in brackets. | | | | | | |  |

Table S9. Model fit of parental intrusiveness (original).

|  | Test statistic |
| --- | --- |
| Model 1 vs. Model 2 | *χ²*(2) = 5.26, *p* = .072 |
| Model 2 vs. Model 3 | *χ²*(1) = 7.06, ***p* = .008** |
| Model 3 vs. Model 4 | *χ²*(1) = 12.10, ***p* = .001** |
| Model 4 vs. Model 5 | *χ²*(2) = 5.87, *p* = .053 |
| Model 5 vs. Model 6 | *χ²*(2) = 0.23, *p* = .892 |

## Multilevel modelling of involvement (CIB).

The assumptions were tested using VIF and tolerance scores to check for multicollinearity (all VIFs were less than 10 and tolerances were greater than 0.01), the Durbin-Watson test to check for dependent residuals (the Durbin-Watson statistic was non-significant, i.e. between 1 and 3) and QQ plots to check for homoscedasticity (visual inspection revealed no issues). We used the lme4 package (Bates et al., 2015) to perform the multilevel analysis.

Table S10. Modelling of children’s involvement.

|  | Model 1 | Model 2 | Model 3 | Model 4 | Model 5 | Model 6 | |
| --- | --- | --- | --- | --- | --- | --- | --- |
| Intercept | 3.486, *p* < .001*** | 3.491, *p* < .001*** | 3.718, *p* < .001*** | 3.708, *p* < .001*** | 3.708, *p* < .001*** | 3.690, *p* < .001*** | |
|  | (0.026) | (0.028) | (0.035) | (0.037) | (0.043) | (0.044) | |
| BPD/ADD vs. CON |  | 0.036, *p* = .063+ | 0.036, *p* = .062+ | 0.031, *p* = .133 | 0.008, *p* = .804 | -0.021, *p* = .538 | |
|  |  | (0.019) | (0.019) | (0.020) | (0.031) | (0.034) | |
| BPD vs. ADD |  | 0.004, *p* = .918 | 0.002, *p* = .947 | -0.005, *p* = .875 | 0.016, *p* = .669 | -0.034, *p* = .472 | |
|  |  | (0.034) | (0.034) | (0.035) | (0.037) | (0.047) | |
| Education low vs. high |  |  |  | 0.026, *p* = .381 | 0.020, *p* = .593 | 0.020, *p* = .591 | |
|  |  |  |  | (0.030) | (0.037) | (0.037) | |
| BPD&ADD vs. CON * low vs. high |  |  |  |  | 0.031, *p* = .313 | 0.031, *p* = .314 | |
|  |  |  |  |  | (0.031) | (0.031) | |
| BPD vs. ADD * low vs. high |  |  |  |  | -0.055, *p* = .144 | -0.055, *p* = .144 | |
|  |  |  |  |  | (0.037) | (0.037) | |
| Free-play vs. structured |  |  | **-0.456, *p* < .001***** | **-0.456, *p* < .001***** | **-0.456, *p* < .001***** | **-0.420, *p* < .001***** | |
|  |  |  | **(0.043)** | **(0.043)** | **(0.043)** | **(0.045)** | |
| BPD&ADD vs. CON * free-play vs. structured |  |  |  |  |  | 0.057, *p* = .071+ | |
|  |  |  |  |  |  | (0.032) | |
| BPD vs. ADD * free-play vs. structured |  |  |  |  |  | 0.099, *p* = .075+ | |
|  |  |  |  |  |  | (0.056) | |
| Num.Obs. | 619 | 619 | 619 | 619 | 619 | 619 | |
| R2 Marg. | 0.000 | 0.006 | 0.137 | 0.138 | 0.142 | 0.151 | |
| R2 Cond. | 0.039 | 0.042 | 0.299 | 0.300 | 0.302 | 0.318 | |
| AIC | 1195.5 | 1206.7 | 1115.1 | 1121.5 | 1132.8 | 1137.3 | |
| BIC | 1208.8 | 1228.9 | 1141.6 | 1152.5 | 1172.6 | 1186.0 | |
| ICC | 0.0 | 0.0 | 0.2 | 0.2 | 0.2 | 0.2 | |
| RMSE | 0.61 | 0.61 | 0.48 | 0.48 | 0.48 | 0.48 | |
| *Note.* + p < .10, * p < .05, ** p < .01, *** p < .001. Standard errors are displayed in brackets. | | | | | | |  |

Table S11. Model fit of children’s involvement.

|  | Test statistic |
| --- | --- |
| Model 1 vs. Model 2 | *χ²*(2) = 3.83, *p* = .147 |
| Model 2 vs. Model 3 | *χ²*(1) = 98.17, ***p* < .001** |
| Model 3 vs. Model 4 | *χ²*(1) = 0.78, *p* = .378 |
| Model 4 vs. Model 5 | *χ²*(2) = 2.69, *p* = .261 |
| Model 5 vs. Model 6 | *χ²*(2) = 8.55, ***p* = .014** |

## Multilevel modelling of withdrawal (CIB).

The assumptions were tested using VIF and tolerance scores to check for multicollinearity (all VIFs were less than 10 and tolerances were greater than 0.01), the Durbin-Watson test to check for dependent residuals (the Durbin-Watson statistic was non-significant, i.e. between 1 and 3) and QQ plots to check for homoscedasticity (visual inspection revealed potential issues). The results should therefore be interpreted with caution. We used the lme4 package (Bates et al., 2015) to perform the multilevel analysis.

Table S12. Modelling of children’s withdrawal.

|  | Model 1 | Model 2 | Model 3 | Model 4 | Model 5 | Model 6 | |
| --- | --- | --- | --- | --- | --- | --- | --- |
| Intercept | 1.438, *p* < .001*** | 1.430, *p* < .001*** | 1.326, *p* < .001*** | 1.336, *p* < .001*** | 1.324, *p* < .001*** | 1.336, *p* < .001*** | |
|  | (0.027) | (0.028) | (0.035) | (0.037) | (0.043) | (0.044) | |
| BPD/ADD vs. CON |  | -0.015, *p* = .462 | -0.015, *p* = .460 | -0.010, *p* = .647 | 0.006, *p* = .861 | 0.009, *p* = .798 | |
|  |  | (0.020) | (0.020) | (0.021) | (0.032) | (0.035) | |
| BPD vs. ADD |  | -0.022, *p* = .534 | -0.021, *p* = .548 | -0.014, *p* = .703 | -0.038, *p* = .321 | 0.001, *p* = .975 | |
|  |  | (0.035) | (0.035) | (0.036) | (0.039) | (0.047) | |
| Education low vs. high |  |  |  | -0.024, *p* = .428 | -0.006, *p* = .882 | -0.006, *p* = .880 | |
|  |  |  |  | (0.031) | (0.039) | (0.039) | |
| BPD&ADD vs. CON * low vs. high |  |  |  |  | -0.020, *p* = .523 | -0.020, *p* = .524 | |
|  |  |  |  |  | (0.032) | (0.032) | |
| BPD vs. ADD * low vs. high |  |  |  |  | 0.068, *p* = .080+ | 0.068, *p* = .0.80+ | |
|  |  |  |  |  | (0.039) | (0.039) | |
| Free-play vs. structured |  |  | **0.207, *p* < .001***** | **0.207, *p* < .001***** | **0.207, *p* < .001***** | **0.182, *p* < .001***** | |
|  |  |  | **(0.040)** | **(0.040)** | **(0.040)** | **(0.042)** | |
| BPD&ADD vs. CON * free-play vs. structured |  |  |  |  |  | -0.007, *p* = .824 | |
|  |  |  |  |  |  | (0.030) | |
| BPD vs. ADD * free-play vs. structured |  |  |  |  |  | -0.080, *p* = .127 | |
|  |  |  |  |  |  | (0.052) | |
| Num.Obs. | 619 | 619 | 619 | 619 | 619 | 619 | |
| R2 Marg. | 0.000 | 0.002 | 0.033 | 0.034 | 0.040 | 0.043 | |
| R2 Cond. | 0.249 | 0.252 | 0.309 | 0.311 | 0.313 | 0.316 | |
| AIC | 1098.0 | 1111.7 | 1092.1 | 1098.6 | 1109.2 | 1119.8 | |
| BIC | 1111.3 | 1133.8 | 1118.7 | 1129.6 | 1149.1 | 1168.5 | |
| ICC | 0.2 | 0.3 | 0.3 | 0.3 | 0.3 | 0.3 | |
| RMSE | 0.46 | 0.46 | 0.43 | 0.43 | 0.43 | 0.43 | |
| *Note.* + p < .10, * p < .05, ** p < .01, *** p < .001. Standard errors are displayed in brackets. | | | | | | |  |

Table S13. Model fit of children’s withdrawal.

|  | Test statistic |
| --- | --- |
| Model 1 vs. Model 2 | *χ²*(2) = 1.24, *p* = .538 |
| Model 2 vs. Model 3 | *χ²*(1) = 26.20, ***p* < .001** |
| Model 3 vs. Model 4 | *χ²*(1) = 0.64, *p* = .425 |
| Model 4 vs. Model 5 | *χ²*(2) = 3.20, *p* = .202 |
| Model 5 vs. Model 6 | *χ²*(2) = 2.74, *p* = .254 |

## Multilevel modelling of reciprocity (CIB).

The assumptions were tested using VIF and tolerance scores to check for multicollinearity (all VIFs were less than 10 and tolerances were greater than 0.01), the Durbin-Watson test to check for dependent residuals (the Durbin-Watson statistic was non-significant, i.e. between 1 and 3) and QQ plots to check for homoscedasticity (visual inspection revealed no issues). We used the lme4 package (Bates et al., 2015) to perform the multilevel analysis.

Table S14. Modelling of dyadic reciprocity.

|  | Model 1 | Model 2 | Model 3 | Model 4 | Model 5 | Model 6 | |
| --- | --- | --- | --- | --- | --- | --- | --- |
| Intercept | 3.629, *p* < .001*** | 3.654, *p* < .001*** | 3.805, *p* < .001*** | 3.753, *p* < .001*** | 3.803, *p* < .001*** | 3.790, *p* < .001*** | |
|  | (0.038) | (0.040) | (0.048) | (0.051) | (0.060) | (0.061) | |
| BPD/ADD vs. CON |  | **0.059, *p* = .034*** | **0.060, *p* = .034*** | 0.032, *p* = .270 | 0.029, *p* = .499 | 0.011, *p* = .825 | |
|  |  | **(0.028)** | **(0.028)** | (0.029) | (0.043) | (0.048) | |
| BPD vs. ADD |  | 0.063, *p* = .198 | 0.062, *p* = .206 | 0.023, *p* = .639 | 0.069, *p* = .192 | 0.030, *p* = .643 | |
|  |  | (0.049) | (0.049) | (0.050) | (0.053) | (0.064) | |
| Education low vs. high |  |  |  | **0.130, *p* = .002**** | 0.069, *p* = .197 | 0.069, *p* = .196 | |
|  |  |  |  | **(0.043)** | (0.053) | (0.053) | |
| BPD&ADD vs. CON * low vs. high |  |  |  |  | 0.003, *p* = .937 | 0.003, *p* = .937 | |
|  |  |  |  |  | (0.043) | (0.043) | |
| BPD vs. ADD * low vs. high |  |  |  |  | **-0.138, *p* = .009**** | **-0.138, *p* = .009**** | |
|  |  |  |  |  | **(0.053)** | **(0.053)** | |
| Free-play vs. structured |  |  | **-0.302, *p* < .001***** | **-0.302, *p* < .001***** | **-0.302, *p* < .001***** | **-0.275, *p* < .001***** | |
|  |  |  | **(0.055)** | **(0.055)** | **(0.055)** | **(0.059)** | |
| BPD&ADD vs. CON * free-play vs. structured |  |  |  |  |  | 0.037, *p* = .369 | |
|  |  |  |  |  |  | (0.041) | |
| BPD vs. ADD * free-play vs. structured |  |  |  |  |  | 0.079, *p* = .276 | |
|  |  |  |  |  |  | (0.072) | |
| Num.Obs. | 619 | 619 | 619 | 619 | 619 | 619 | |
| R2 Marg. | 0.000 | 0.016 | 0.049 | 0.067 | 0.080 | 0.083 | |
| R2 Cond. | 0.264 | 0.268 | 0.330 | 0.331 | 0.333 | 0.335 | |
| AIC | 1517.4 | 1523.1 | 1500.1 | 1497.3 | 1502.9 | 1512.2 | |
| BIC | 1530.7 | 1545.2 | 1526.6 | 1528.3 | 1542.7 | 1560.9 | |
| ICC | 0.3 | 0.3 | 0.3 | 0.3 | 0.3 | 0.3 | |
| RMSE | 0.63 | 0.64 | 0.60 | 0.60 | 0.60 | 0.60 | |
| *Note.* + p < .10, * p < .05, ** p < .01, *** p < .001. Standard errors are displayed in brackets. | | | | | | |  |

Table S15. Model fit of dyadic reciprocity.

|  | Test statistic |
| --- | --- |
| Model 1 vs. Model 2 | *χ²*(2) = 7.97, ***p* = .019** |
| Model 2 vs. Model 3 | *χ²*(1) = 28.96, ***p* < .001** |
| Model 3 vs. Model 4 | *χ²*(1) = 9.32, ***p* = .002** |
| Model 4 vs. Model 5 | *χ²*(2) = 7.08, ***p* = .029** |
| Model 5 vs. Model 6 | *χ²*(2) = 2.67, *p* = .263 |

## Multilevel modelling of negative states (CIB).

The assumptions were tested using VIF and tolerance scores to check for multicollinearity (all VIFs were less than 10 and tolerances were greater than 0.01), the Durbin-Watson test to check for dependent residuals (the Durbin-Watson statistic was non-significant, i.e. between 1 and 3) and QQ plots to check for homoscedasticity (visual inspection revealed potential issues). The results should therefore be interpreted with caution. We used the lme4 package (Bates et al., 2015) to perform the multilevel analysis.

Table S16. Modelling of dyadic negative states.

|  | Model 1 | Model 2 | Model 3 | Model 4 | Model 5 | Model 6 | |
| --- | --- | --- | --- | --- | --- | --- | --- |
| Intercept | 1.681, *p* < .001*** | 1.656, *p* < .001*** | 1.551, *p* < .001*** | 1.598, *p* < .001*** | 1.573, *p* < .001*** | 1.581, *p* < .001*** | |
|  | (0.034) | (0.036) | (0.046) | (0.048) | (0.056) | (0.057) | |
| BPD/ADD vs. CON |  | -0.048, *p* = .062+ | -0.048, *p* = .061+ | -0.023, *p* = .381 | -0.006, *p* = .888 | 0.016, *p* = .716 | |
|  |  | (0.026) | (0.026) | (0.027) | (0.040) | (0.045) | |
| BPD vs. ADD |  | -0.064, *p* = .151 | -0.064, *p* = .156 | -0.029, *p* = .526 | -0.067, *p* = .172 | -0.048, *p* = .432 | |
|  |  | (0.045) | (0.045) | (0.046) | (0.049) | (0.061) | |
| Education low vs. high |  |  |  | **-0.117, *p* = .003**** | -0.082, *p* = .091+ | -0.082, *p* = .091+ | |
|  |  |  |  | **(0.039)** | (0.049) | (0.049) | |
| BPD&ADD vs. CON * low vs. high |  |  |  |  | -0.023, *p* = .555 | -0.023, *p* = .555 | |
|  |  |  |  |  | (0.040) | (0.040) | |
| BPD vs. ADD * low vs. high |  |  |  |  | **0.106, *p* = .029*** | **0.106, *p* = .029*** | |
|  |  |  |  |  | **(0.049)** | **(0.049)** | |
| Free-play vs. structured |  |  | **0.210, *p* < .001***** | **0.210, *p* < .001***** | **0.211, *p* < .001***** | **0.195, *p* = .001**** | |
|  |  |  | **(0.055)** | **(0.055)** | **(0.055)** | **(0.059)** | |
| BPD&ADD vs. CON * free-play vs. structured |  |  |  |  |  | -0.044, *p* = .295 | |
|  |  |  |  |  |  | (0.042) | |
| BPD vs. ADD * free-play vs. structured |  |  |  |  |  | -0.037, *p* = .611 | |
|  |  |  |  |  |  | (0.073) | |
| Num.Obs. | 619 | 619 | 619 | 619 | 619 | 619 | |
| R2 Marg. | 0.000 | 0.014 | 0.032 | 0.048 | 0.057 | 0.059 | |
| R2 Cond. | 0.190 | 0.194 | 0.228 | 0.228 | 0.231 | 0.232 | |
| AIC | 1451.1 | 1457.7 | 1449.5 | 1447.2 | 1455.3 | 1465.6 | |
| BIC | 1464.3 | 1479.8 | 1476.1 | 1478.2 | 1495.2 | 1514.3 | |
| ICC | 0.2 | 0.2 | 0.2 | 0.2 | 0.2 | 0.2 | |
| RMSE | 0.64 | 0.65 | 0.63 | 0.63 | 0.63 | 0.63 | |
| *Note.* + p < .10, * p < .05, ** p < .01, *** p < .001. Standard errors are displayed in brackets. | | | | | | |  |

Table S17. Model fit of dyadic negative states.

|  | Test statistic |
| --- | --- |
| Model 1 vs. Model 2 | *χ²*(2) = 7.34, ***p* = .025** |
| Model 2 vs. Model 3 | *χ²*(1) = 14.14, ***p* < .001** |
| Model 3 vs. Model 4 | *χ²*(1) = 9.00, ***p* = .003** |
| Model 4 vs. Model 5 | *χ²*(2) = 4.83, ***p* = .089** |
| Model 5 vs. Model 6 | *χ²*(2) = 1.75, *p* = .418 |

## Multilevel modelling of observed behavior and child age groups (CIB).

Table S18. Observed maternal, child, and dyadic behavior (CIB) per group, child age, and task.

|  | BPD | | | | ADD | | | | CON | | | |
| --- | --- | --- | --- | --- | --- | --- | --- | --- | --- | --- | --- | --- |
|  | younger | | older | | younger | | older | | younger | | older | |
|  | free | struc | free | struc | free | struc | free | struc | free | struc | free | struc |
| N | 65 | 65 | 89 | 91 | 30 | 30 | 33 | 32 | 57 | 57 | 35 | 35 |
| CIB: Sensitivity (mean/SD) | 3.529 (0.620) | 3.355 (0.726) | 3.557 (0.577) | 3.199 (0.563) | 3.617 (0.417) | 3.469 (0.468) | 3.612 (0.509) | 3.280 (0.605) | 3.715 (0.440) | 3.589 (0.446) | 3.540 (0.474) | 3.549 (0.497) |
| CIB: Intrusiveness (mean/SD) | 1.625 (0.669) | 1.662 (0.642) | 1.266 (0.354) | 1.376 (0.464) | 1.344 (0.329) | 1.475 (0.361) | 1.224 (0.298) | 1.351 (0.448) | 1.354 (0.390) | 1.512 (0.450) | 1.174 (0.374) | 1.155 (0.186) |
| CIB: Involvement (mean/SD) | 3.315 (0.615) | 3.279 (0.604) | 4.053 (0.531) | 3.083 (0.490) | 3.284 (0.444) | 3.349 (0.649) | 3.975 (0.448) | 3.189 (0.493) | 3.550 (0.558) | 3.451 (0.557) | 3.986 (0.460) | 3.343 (0.465) |
| CIB: Withdrawal (mean/SD) | 1.549 (0.620) | 1.729 (0.744) | 1.166 (0.305) | 1.508 (0.607) | 1.612 (0.743) | 1.660 (0.699) | 1.148 (0.327) | 1.310 (0.399) | 1.389 (0.566) | 1.569 (0.643) | 1.196 (0.341) | 1.348 (0.508) |
| CIB: Reciprocity (mean/SD) | 3.492 (0.845) | 3.331 (0.966) | 3.911 (0.820) | 3.343 (0.845) | 3.446 (0.667) | 3.554 (0.636) | 4.071 (0.744) | 3.529 (0.789) | 3.860 (0.706) | 3.573 (0.858) | 3.895 (0.693) | 3.836 (0.674) |
| CIB: Negative States (mean/SD) | 1.800 (0.879) | 1.935 (0.948) | 1.500 (0.700) | 1.885 (0.851) | 1.726 (0.732) | 1.644 (0.653) | 1.371 (0.534) | 1.827 (0.870) | 1.520 (0.669) | 1.662 (0.784) | 1.486 (0.581) | 1.536 (0.588) |
| *Note.* Younger = Children < 36 months (i.e., 3 years), Older = Children $\geq$ 36 months (i.e., 3 years), Free = free-play, Struc = structured play. | | | | | | | | | | | | |

### Parental sensitivity.

The assumptions were tested using VIF and tolerance scores to check for multicollinearity (all VIFs were less than 10 and tolerances were greater than 0.01), the Durbin-Watson test to check for dependent residuals (the Durbin-Watson statistic was non-significant, i.e. between 1 and 3) and QQ plots to check for homoscedasticity (visual inspection revealed potential issues). The results should therefore be interpreted with caution. We used the lme4 package (Bates et al., 2015) to perform the multilevel analysis.

Mothers with younger children (mean = 3.54, SD = 0.56) were equally sensitive than mothers with older children (mean = 3.43, SD = 0.57).

Table S19. Modelling of parental sensitivity, including dichotomized child age as a predictor.

|  | Model 1: Null | Model 4 |
| --- | --- | --- |
| Intercept | 3.482, *p* < .001*** | 3.609, *p* < .001*** |
|  | (0.026) | (0.043) |
| BPD/ADD vs. CON |  | 0.032, *p* = .107 |
|  |  | (0.020) |
| BPD vs. ADD |  | 0.016, *p* = .633 |
|  |  | (0.034) |
| Children: younger vs. older |  | -0.076, *p* = .135 |
|  |  | (0.051) |
| Education low vs. high |  | **0.088, *p* = .002**** |
|  |  | **(0.029)** |
| Free-play vs. structured |  | **-0.210, *p* < .001***** |
|  |  | **(0.038)** |
| Num.Obs. | 619 | 619 |
| R2 Marg. | 0.000 | 0.082 |
| R2 Cond. | 0.259 | 0.331 |
| AIC | 1049.9 | 1030.6 |
| BIC | 1063.2 | 1066.0 |
| ICC | 0.3 | 0.3 |
| RMSE | 0.44 | 0.41 |
| *Note.* + p < .10, * p < .05, ** p < .01, *** p < .001. Standard errors are displayed in brackets. Model fit between model 1 vs. model 4: $\chi^{2}$(5) = 54.70, *p* < .001***. | | |

### Parental intrusiveness.

The assumptions were tested using VIF and tolerance scores to check for multicollinearity (all VIFs were less than 10 and tolerances were greater than 0.01), the Durbin-Watson test to check for dependent residuals (the Durbin-Watson statistic was non-significant, i.e. between 1 and 3) and QQ plots to check for homoscedasticity (visual inspection revealed potential issues). The results should therefore be interpreted with caution. We used the lme4 package (Bates et al., 2015) to perform the multilevel analysis.

Mothers with younger children (mean = 1.52, SD = 0.53) were more intrusive than mothers with older children (mean = 1.28, SD = 0.39).

Table S20. Modelling of parental intrusiveness, including dichotomized child age as a predictor.

|  | Model 1: Null | Model 4 |
| --- | --- | --- |
| Intercept | 1.397, *p* < .001*** | 1.512, *p* < .001*** |
|  | (0.023) | (0.035) |
| BPD/ADD vs. CON |  | -0.017, *p* = .299 |
|  |  | (0.016) |
| BPD vs. ADD |  | -0.033, *p* = .242 |
|  |  | (0.028) |
| Children: younger vs. older |  | **-0.277, *p* < .001***** |
|  |  | **(0.042)** |
| Education low vs. high |  | **-0.105, *p* < .001***** |
|  |  | **(0.024)** |
| Free-play vs. structured |  | **0.092, *p* = .003**** |
|  |  | **(0.030)** |
| Num.Obs. | 619 | 619 |
| R2 Marg. | 0.000 | 0.133 |
| R2 Cond. | 0.365 | 0.385 |
| AIC | 817.0 | 782.8 |
| BIC | 830.3 | 818.2 |
| ICC | 0.4 | 0.3 |
| RMSE | 0.33 | 0.33 |
| *Note.* + p < .10, * p < .05, ** p < .01, *** p < .001. Standard errors are displayed in brackets. Model fit between model 1 vs. model 4: $\chi^{2}$(5) = 71.66, *p* < .001***. | | |

### Child involvement.

The assumptions were tested using VIF and tolerance scores to check for multicollinearity (all VIFs were less than 10 and tolerances were greater than 0.01), the Durbin-Watson test to check for dependent residuals (the Durbin-Watson statistic was non-significant, i.e. between 1 and 3) and QQ plots to check for homoscedasticity (visual inspection indicated homoscedasticity). We used the lme4 package (Bates et al., 2015) to perform the multilevel analysis.

Younger children (mean = 3.38, SD = 0.58) were less involved than older children (mean = 3.59, SD = 0.66).

Table S21. Modelling of child involvement, including dichotomized child age as a predictor.

|  | Model 1: Null | Model 4 |
| --- | --- | --- |
| Intercept | 3.486, *p* < .001*** | 3.585, *p* < .001*** |
|  | (0.026) | (0.044) |
| BPD/ADD vs. CON |  | **0.042, *p* = .033*** |
|  |  | **(0.020)** |
| BPD vs. ADD |  | -0.001, *p* = .975 |
|  |  | (0.034) |
| Children: younger vs. older |  | **0.242, *p* < .001***** |
|  |  | **(0.051)** |
| Education low vs. high |  | 0.035, *p* = .219 |
|  |  | (0.029) |
| Free-play vs. structured |  | **-0.456, *p* < .001***** |
|  |  | **(0.043)** |
| Num.Obs. | 619 | 619 |
| R2 Marg. | 0.000 | 0.173 |
| R2 Cond. | 0.039 | 0.301 |
| AIC | 1195.5 | 1105.5 |
| BIC | 1208.8 | 1140.9 |
| ICC | 0.0 | 0.2 |
| RMSE | 0.61 | 0.49 |
| *Note.* + p < .10, * p < .05, ** p < .01, *** p < .001. Standard errors are displayed in brackets. Model fit between model 1 vs. model 4: $\chi^{2}$(5) = 125.21, *p* < .001***. | | |

### Child withdrawal.

The assumptions were tested using VIF and tolerance scores to check for multicollinearity (all VIFs were less than 10 and tolerances were greater than 0.01), the Durbin-Watson test to check for dependent residuals (the Durbin-Watson statistic was non-significant, i.e. between 1 and 3) and QQ plots to check for homoscedasticity (visual inspection revealed potential issues). The results should therefore be interpreted with caution. We used the lme4 package (Bates et al., 2015) to perform the multilevel analysis.

Younger children (mean = 1.58, SD = 0.67) were more withdrawn than older children (mean = 1.30, SD = 0.47).

Table S22. Modelling of child withdrawal, including dichotomized child age as a predictor.

|  | Model 1: Null | Model 4 |
| --- | --- | --- |
| Intercept | 1.438, *p* < .001*** | 1.488, *p* < .001*** |
|  | (0.027) | (0.044) |
| BPD/ADD vs. CON |  | -0.024, *p* = .231 |
|  |  | (0.020) |
| BPD vs. ADD |  | -0.019, *p* = .577 |
|  |  | (0.034) |
| Children: younger vs. older |  | **-0.299, *p* < .001***** |
|  |  | **(0.051)** |
| Education low vs. high |  | -0.036, *p* = .219 |
|  |  | (0.029) |
| Free-play vs. structured |  | **0.207, *p* < .001***** |
|  |  | **(0.040)** |
| Num.Obs. | 619 | 619 |
| R2 Marg. | 0.000 | 0.095 |
| R2 Cond. | 0.249 | 0.310 |
| AIC | 1098.0 | 1072.5 |
| BIC | 1111.3 | 1107.9 |
| ICC | 0.2 | 0.2 |
| RMSE | 0.46 | 0.44 |
| *Note.* + p < .10, * p < .05, ** p < .01, *** p < .001. Standard errors are displayed in brackets. Model fit between model 1 vs. model 4: $\chi^{2}$(5) = 60.69, *p* < .001***. | | |

### Dyadic reciprocity.

The assumptions were tested using VIF and tolerance scores to check for multicollinearity (all VIFs were less than 10 and tolerances were greater than 0.01), the Durbin-Watson test to check for dependent residuals (the Durbin-Watson statistic was non-significant, i.e. between 1 and 3) and QQ plots to check for homoscedasticity (visual inspection indicated homoscedasticity). We used the lme4 package (Bates et al., 2015) to perform the multilevel analysis.

Dyads with younger children (mean = 3.54, SD = 0.83) were less reciprocal than dyads with older children (mean = 3.71, SD = 0.83).

Table S23. Modelling of dyadic reciprocity, including dichotomized child age as a predictor.

|  | Model 1: Null | Model 4 |
| --- | --- | --- |
| Intercept | 3.629, *p* < .001*** | 3.637, *p* < .001*** |
|  | (0.038) | (0.063) |
| BPD/ADD vs. CON |  | 0.043, *p* = .136 |
|  |  | (0.029) |
| BPD vs. ADD |  | 0.028, *p* = .575 |
|  |  | (0.049) |
| Children: younger vs. older |  | **0.227, *p* = .002**** |
|  |  | **(0.074)** |
| Education low vs. high |  | **0.139, *p* = .001**** |
|  |  | **(0.042)** |
| Free-play vs. structured |  | **-0.302, *p* < .001***** |
|  |  | **(0.055)** |
| Num.Obs. | 619 | 619 |
| R2 Marg. | 0.000 | 0.084 |
| R2 Cond. | 0.264 | 0.331 |
| AIC | 1517.4 | 1493.3 |
| BIC | 1530.7 | 1528.8 |
| ICC | 0.3 | 0.3 |
| RMSE | 0.63 | 0.60 |
| *Note.* + p < .10, * p < .05, ** p < .01, *** p < .001. Standard errors are displayed in brackets. Model fit between model 1 vs. model 4: $\chi^{2}$(5) = 55.74, *p* < .001***. | | |

### Dyadic negative states.

The assumptions were tested using VIF and tolerance scores to check for multicollinearity (all VIFs were less than 10 and tolerances were greater than 0.01), the Durbin-Watson test to check for dependent residuals (the Durbin-Watson statistic was non-significant, i.e. between 1 and 3) and QQ plots to check for homoscedasticity (visual inspection revealed potential issues). The results should therefore be interpreted with caution. We used the lme4 package (Bates et al., 2015) to perform the multilevel analysis.

Dyads with younger children (mean = 1.73, SD = 0.81) engaged in more negative states than dyads with older children (mean = 1.63, SD = 0.75).

Table S24. Modelling of dyadic negative states, including dichotomized child age as a predictor.

|  | Model 1: Null | Model 4 |
| --- | --- | --- |
| Intercept | 1.681, *p* < .001*** | 1.671, *p* < .001*** |
|  | (0.034) | (0.059) |
| BPD/ADD vs. CON |  | -0.030, *p* = .257 |
|  |  | (0.027) |
| BPD vs. ADD |  | -0.032, *p* = .487 |
|  |  | (0.045) |
| Children: younger vs. older |  | **-0.142, *p* = .037*** |
|  |  | **(0.068)** |
| Education low vs. high |  | **-0.122, *p* = .002**** |
|  |  | **(0.039)** |
| Free-play vs. structured |  | **0.210, *p* < .001***** |
|  |  | **(0.055)** |
| Num.Obs. | 619 | 619 |
| R2 Marg. | 0.000 | 0.056 |
| R2 Cond. | 0.190 | 0.229 |
| AIC | 1451.1 | 1448.5 |
| BIC | 1464.3 | 1483.9 |
| ICC | 0.2 | 0.2 |
| RMSE | 0.64 | 0.63 |
| *Note.* + p < .10, * p < .05, ** p < .01, *** p < .001. Standard errors are displayed in brackets. Model fit between model 1 vs. model 4: $\chi^{2}$(5) = 34.87, *p* < .001***. | | |

## Two-way independent ANOVAs (PRQ, CRBI).

The assumption of independent variables and interval-scaled dependent variables was met in all models. Variance homogeneity was tested separately for each model using the Levene test, and normal distribution within the groups was tested using the Shapiro–Wilk test, bootstrapped confidence intervals of the group means and QQ plots. As the Shapiro-Wilk test is highly sensitive in larger samples and provides significant results more quickly (Field et al., 2012), we relied mostly on visual inspection.

For the PRQ Attachment scale, the assumption of variance homogeneity was not met (group: *F*(2, 216) = 5.532, *p* = .005, education: *F*(1, 217) = 1.654, *p* = .200, group*education: *F*(5, 213) = 2.452, *p* = .035). The Shapiro-Wilk test revealed issues with the normal distribution (*W* = 0.98, *p* = .004), but visual inspection of the bootstrapped CIs and the QQ plot did not. Due to the heterogeneity and the problems with the normal distribution, we relied on a robust ANOVA using M-measures of the median and bootstrapping (using the *WRS2* package, Mair & Wilcox, 2020)*.*

The assumptions of variance homogeneity were met for the PRQ Involvement scale (group: *F*(2, 217) = 0.625, *p* = .536, education: *F*(1, 218) = 0.096, *p* = .758, group*education: *F*(5, 214) = 0.546, *p* = .741). Issues with the normal distribution were indicated by the Shapiro Wilk test (*W* = 0.982, *p* = .006), but neither by the bootstrapped CIs nor the QQ plot.

The PRQ Relational Frustration scale met the assumptions of variance homogeneity (group: *F*(2, 217) = 2.713, *p* = .069, education: *F*(1, 218) = 0.513, *p* = .475, group*education: *F*(5, 214) = 1.742, *p* = .126). Both the Shapiro-Wil test (*W* = 0.979, *p* = .002) and the bootstrapped CI figure indicated problems with the normal distribution. The QQ plot, however, did not.

The CRBI met the assumptions of variance homogeneity (group: *F*(2, 215) = 2.016, *p* = .136, education: *F*(1, 216) = 0.03, *p* = .862, group*education: *F*(5, 212) = 1.344, *p* = .247). While the Shapiro-Wilk test indicated potential problems with the normal distribution (*W* = 0.913, *p* < .001), the visual inspection of the bootstrapped CIs and the QQ plot did not.

Table S25. Results of two-way independent ANOVAs with group and education as predictors.

|  | N | Group | Education | Group * Education | Contrast:  BPD & AD/D vs. CON | Contrast:  BPD vs. AD/D |
| --- | --- | --- | --- | --- | --- | --- |
| PRQ: Attachment^1,2^ | 219 | ***p* < .001** | *p* = .607 | *p* = .607 | $\hat{\Psi}$ = -14.348, ***p* = .042** | $\hat{\Psi}$ = -7.837, *p* = .127 |
| PRQ: Involvement | 220 | *F*(2, 214) = 4.192,  ***p* = .016** | *F*(1, 214) = 1.528,  *p* = .218 | *F*(2, 214) = 0.064,  *p* = .938 | *t*(214) = 2.697, ***p* = .008**,  *d* = -0.70, *r* = -0.30 | *t*(214) = 0.581, *p* = .562,  *d* = -0.16, *r* = -0.07 |
| PRQ: Relational Frustration | 220 | *F*(2, 214) = 20.964, ***p* < .001** | *F*(1, 214) = 0.328,  *p* = .567 | *F*(2, 214) = 0.576,  *p* = .563 | *t*(214) = -5.04, ***p* < .001**,  *d* = 1.31, *r* = 0.51 | *t*(214) = -3.152, ***p* = .002**, *d* = 0.56, *r* = 0.25 |
| CRBI | 218 | *F*(2, 212) = 7.672,  ***p* = .001** | *F*(1, 212) = 0.65,  *p* = .421 | *F*(2, 212) = 0.866,  *p* = .422 | *t*(212) = 3.391, ***p* = .001**,  *d* = -0.67, *r* = -0.29 | *t*(212) = 1.38, *p* = .169,  *d* = -0.29, *r* = -0.13 |
| *Note.* ^1^One missing value in PRQ Attachment. ^2^When assumptions where violated (especially the assumption of variance homogeneity), we performed robust ANOVAs. BPD: mothers with borderline personality disorder, AD/D: mothers with an anxiety disorder, depression, or both, CON: mothers without any mental disorder. | | | | | | |
